# OLB-AC: toward optimizing ligand bioactivities through deep graph learning and activity cliffs

**DOI:** 10.1093/bioinformatics/btae365

**Published:** 2024-06-18

**Authors:** Yueming Yin, Haifeng Hu, Jitao Yang, Chun Ye, Wilson Wen Bin Goh, Adams Wai-Kin Kong, Jiansheng Wu

**Affiliations:** School of Telecommunications and Information Engineering, Nanjing University of Posts and Telecommunications, Nanjing 210003, China; College of Computing and Data Science, Nanyang Technological University, 639798, Singapore; School of Telecommunications and Information Engineering, Nanjing University of Posts and Telecommunications, Nanjing 210003, China; School of Telecommunications and Information Engineering, Nanjing University of Posts and Telecommunications, Nanjing 210003, China; School of Telecommunications and Information Engineering, Nanjing University of Posts and Telecommunications, Nanjing 210003, China; Lee Kong Chian School of Medicine, Nanyang Technological University, 637551, Singapore; School of Biological Sciences, Nanyang Technological University, 637551, Singapore; Center for Biomedical Informatics, Nanyang Technological University, 637551, Singapore; Center for AI in Medicine, Nanyang Technological University, 639798, Singapore; Division of Neurology, Department of Brain Sciences, Faculty of Medicine, Imperial College London, London W12 0NN, U.K; College of Computing and Data Science, Nanyang Technological University, 639798, Singapore; School of Computer Science, Nanjing University of Posts and Telecommunications, Nanjing 210023, China

## Abstract

**Motivation:**

Deep graph learning (DGL) has been widely employed in the realm of ligand-based virtual screening. Within this field, a key hurdle is the existence of activity cliffs (ACs), where minor chemical alterations can lead to significant changes in bioactivity. In response, several DGL models have been developed to enhance ligand bioactivity prediction in the presence of ACs. Yet, there remains a largely unexplored opportunity within ACs for optimizing ligand bioactivity, making it an area ripe for further investigation.

**Results:**

We present a novel approach to simultaneously predict and optimize ligand bioactivities through DGL and ACs (OLB-AC). OLB-AC possesses the capability to optimize ligand molecules located near ACs, providing a direct reference for optimizing ligand bioactivities with the matching of original ligands. To accomplish this, a novel attentive graph reconstruction neural network and ligand optimization scheme are proposed. Attentive graph reconstruction neural network reconstructs original ligands and optimizes them through adversarial representations derived from their bioactivity prediction process. Experimental results on nine drug targets reveal that out of the 667 molecules generated through OLB-AC optimization on datasets comprising 974 low-activity, noninhibitor, or highly toxic ligands, 49 are recognized as known highly active, inhibitor, or nontoxic ligands beyond the datasets’ scope. The 27 out of 49 matched molecular pairs generated by OLB-AC reveal novel transformations not present in their training sets. The adversarial representations employed for ligand optimization originate from the gradients of bioactivity predictions. Therefore, we also assess OLB-AC’s prediction accuracy across 33 different bioactivity datasets. Results show that OLB-AC achieves the best Pearson correlation coefficient (*r*^2^) on 27/33 datasets, with an average improvement of 7.2%–22.9% against the state-of-the-art bioactivity prediction methods.

**Availability and implementation:**

The code and dataset developed in this work are available at github.com/Yueming-Yin/OLB-AC.

## 1 Introduction

Ligands can be naturally represented as molecular graphs at the atom level, where nodes represent atoms and edges denote bonds (Rong *et al.* 2020, Ma *et al.* 2020, Li *et al.* 2021, Shi *et al.* 2022, Sánchez-Cruz 2023). Compared to typical sequence-based methods that operate on the SMILES format, graph-based algorithms have an advantage in uncovering the hidden relationships among molecular atoms and bonds (Li *et al.* 2022b). Ligand bioactivities are typically evaluated by the IC50, EC50, Ki, and Kd values (Musumeci *et al.* 2017, Jabeen and Ranganathan 2019, Yang *et al.* 2021). Accurate assessment of ligand bioactivities is a crucial aspect of drug discovery, which starts with screening extensive chemical compound databases against target proteins via high-throughput assays (Sadybekov and Katritch 2023). Lead hits are selected based on their bioactivity and optimized to improve their inhibitory properties for drug targets (Sawada *et al.* 2018). High-throughput assays for screening molecular compounds are time- and labor-intensive, thus computational virtual screening methods have become a fundamental supplementation of experimental approaches. Computational pipelines that accurately model the bioactivities of ligand molecules are necessary for virtual screening and optimizing hit compounds (Wu *et al.* 2019).

At present, many deep graph learning (DGL) models have been proposed and utilized for ligand bioactivity prediction or ligand-based virtual screening. For instance, Duvenaud *et al.* 2015 proposed a graph convolutional network called Neural FPs, enabling end-to-end learning of molecular fingerprints from graphs of arbitrary size and shape, and applied it to the prediction of drug bioactivities and other properties. He *et al.* (2021b) aimed to address the urgent need for new bioactive ligands in the treatment of breast cancer by utilizing DGL techniques. Li *et al.* (2022a) proposed KPGT, a new knowledge-guided pre-training Graph Transformer model, a novel self-supervised learning framework for molecular graph representation learning. Zhou *et al.* (2023) deployed a universal molecular representation learning framework that enlarges the representation ability and application scope of molecular representation learning schemes including ligand bioactivity prediction.

Activity cliffs (ACs) are often characterized as pairs or groups of structurally similar ligand molecules that exhibit bioactivity against the same target but display significant differences in their activity levels (Dimova and Bajorath 2016). ACs inherently violate the assumptions of the quantitative structure–activity relationship (QSAR) premise, which “form a major source of prediction error in QSAR models” as concluded by Dablander *et al.* (2023). Currently, there are several research efforts focused on developing novel DGL methods to mitigate the impact of ACs on the performance of bioactivity prediction. For example, Kireeva *et al.* (2014) demonstrated the effectiveness of distance-based metric learning procedures in reducing the number of ACs. Winkler and Le (2017) investigated how deep neural networks can address challenges posed by ACs in QSAR datasets. Shen *et al.* 2023 introduced a new online triplet contrastive learning framework (ACANet) to enable efficient AC awareness in molecular activity prediction. Yin *et al.* (2022a) proposed AFSE to improve generalization performance and reduce the impact of ACs on bioactivity prediction. They minimized the maximum prediction discrepancy on perturbed molecular features in a feature subspace. The resulting adversarial perturbations are used as clues for molecular optimization in this paper.

This natural phenomenon, known as ACs, involves pairs of structurally similar molecules with notable differences in bioactivity. Could we use this natural phenomenon to provide valuable information to improve the bioactivity of ligand molecules? The answer is yes. As discussed in Bajorath (2017), the AC problem represents one of the most complex and crucial factors influencing the screening and optimization of lead compounds, which are ligands possessing the appropriate bioactivity and properties for use as drug candidates. Current methods for optimizing ligand bioactivity using ACs mainly focus on predicting existing ACs in databases, providing references for the optimization of ligand bioactivity (Stumpfe *et al.* 2014). For example, Iqbal *et al.* (2021) predicted whether a pair of structural analogs forms an AC or not from image data in different compound activity classes. Dablander *et al.* (2023) systematically investigated the AC-prediction power of modern QSAR methods and its quantitative relationship to general QSAR prediction performance. Their findings highlight the frequent inadequacy of QSAR models in predicting AC, suggesting that enhancing AC sensitivity could be pivotal for improvement. Stumpfe *et al.* (2013) introduced a compound pathway model and determined AC-dependent and -independent pathways for mimic compound optimization. However, determining how to enhance the bioactivity of candidate ligands with reference to known ACs remains a challenging issue, especially when constrained by the ACs present in the database.

Therefore, this paper introduces a novel approach for optimizing ligand bioactivity through ACs, denoted as OLB-AC, that leverages DGL and adversarial representations in its prediction process to infer the matched molecular pairs around ACs (MMP-Cliffs) directly on candidate ligands. OLB-AC comprises a graph embedding network, a new attentive graph reconstruction neural network (AGRN), a newly designed logic for optimizing ligand bioactivity, and the AFSE algorithm (Yin *et al.* 2022a). To establish a foundational understanding of molecular structures and ligand bioactivity, OLB-AC initially learns bioactivity prediction and molecular graph reconstruction on ligand-based virtual screening datasets. Simultaneously, OLB-AC employs the AFSE algorithm to dynamically generate adversarial representations in a new feature subspace. These generated representations manifest a significant shift in model predictions, aiding in ligand optimization on bioactivity. Subsequently, OLB-AC decodes these generated representations to optimize input ligands, enhancing their bioactivity while preserving structural similarity. Finally, OLB-AC adjusts its optimization strategy based on the chemical validity of the optimized ligands.

To validate the effectiveness of optimizing ligand bioactivity in the context of ACs, we selected MMP-Cliffs from the bioactivity database constructed by Yin *et al.* (2022a). In MMP-Cliffs, molecules exhibit small structural differences but have bioactivity values that differ significantly. Results on nine drug targets demonstrate that OLB-AC successfully optimizes ligands, enhancing their bioactivity from low activity to high activity near MMP-Cliffs. Among the 241 molecules generated through OLB-AC optimization on datasets containing 356 low-activity ligands, 17 are identified as known highly active ligands beyond the datasets’ scope. And five of them reveal novel transformations not present in their training sets. Additionally, we deployed OLB-AC on molecular property datasets constructed by Xiong *et al.* (2021) and found that OLB-AC can enhance the inhibitory efficacy of ligands and reduce ligand toxicity through optimizing bioactivity. Among the 426 molecules generated by OLB-AC optimization on datasets containing 618 noninhibitor or toxic ligands, 32 are known inhibitor or nontoxic ligands beyond the datasets’ scope. And 22 of them reveal novel transformations. Furthermore, to ensure the accuracy of OLB-AC in modeling bioactivity, we tested OLB-AC’s bioactivity predictions on 33 bioactivity benchmarks constructed by Yin *et al.* (2022a) using the square of the Pearson correlation coefficient (*r*^2^) index. The results reveal that OLB-AC achieves the best performance on 27 out of 33 datasets, improving *r*^2^ by 7.19% to 22.93% compared to state-of-the-art ligand bioactivity prediction methods.

## 2 Materials and methods

**Figure 1.**
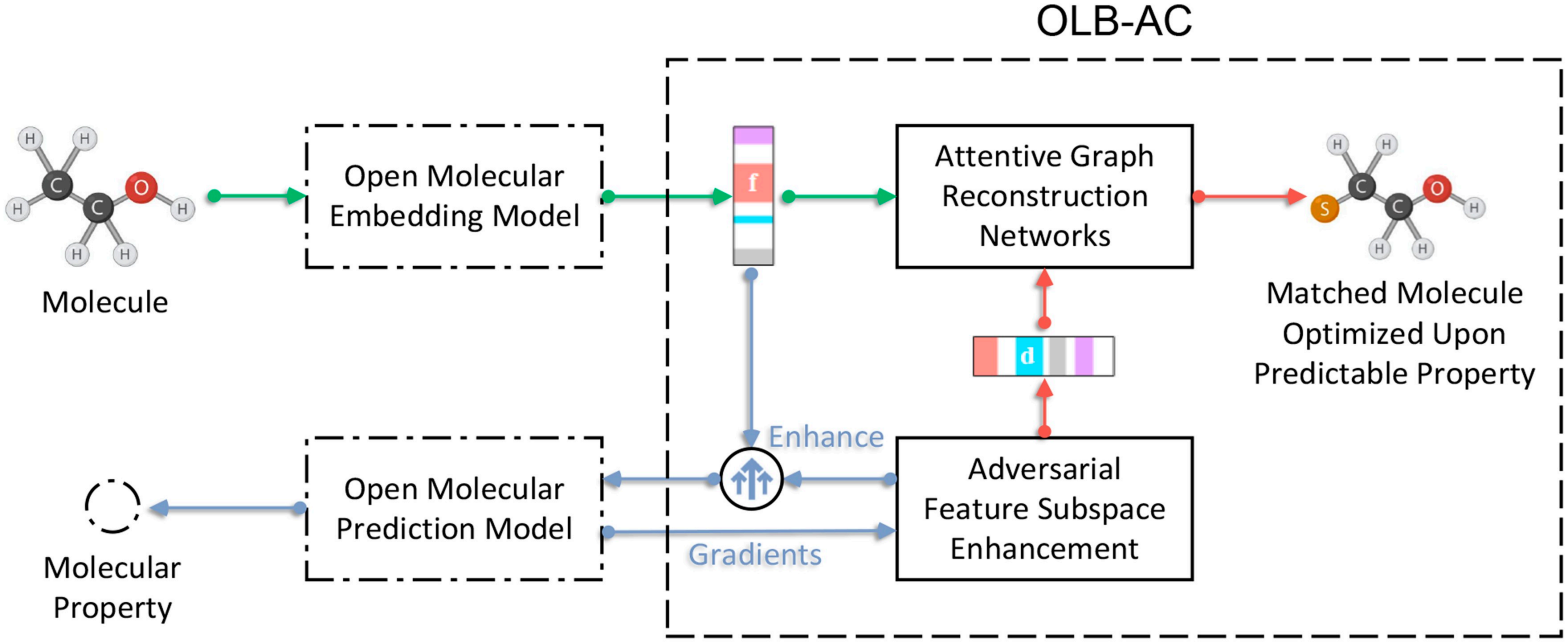
Depiction of OLB-AC’s architecture in molecular prediction pipeline.

**Overview:** Figure 1 depicts the architecture of the OLB-AC algorithm in a general molecular property prediction (MP) pipeline. In Fig. 1, the molecular embedding model [in this paper, we use Attentive FP (Xiong *et al.* 2019) as an example, see Supplementary Algorithm S1 for its implementation] generates molecular embeddings **f** which captures the key molecular graph features and feeds them into OLB-AC for the reconstruction of the input molecules (Fig. 1: green lines). Then, the MP model fits the relationship between molecular embeddings and property values using fully connected neural networks and provides its gradient on molecular embeddings to OLB-AC (Fig. 1: input blue line to OLB-AC). Inside OLB-AC, the AFSE (Yin *et al.* 2022a) algorithm (Section 2.1.1) generates adversarial perturbations **d** based on the molecular embedding gradient and uses them to enhance the generalization of the MP model (Fig. 1: output blue line from OLB-AC). Meanwhile, an AGRN (Section 2.1) reconstructs (Section 2.2) and optimizes (Sections 2.3.2 and 2.3.3) molecule upon the predictable property according to the molecular embedding **f** and the adversarial perturbation **d** (Fig. 1: red lines). The total objective of OLB-AC is shown in Section 2.3.3 We summarized all notations in Supplementary Table S1.

**Subsection arrangement:** Section 2.1 is dedicated to constructing an AGRN for ligand graph generation. AGRN achieves this by seeking cues for molecular optimization, breaking down molecular embeddings, and facilitating message passing from molecules to atoms and from atoms to neighboring atoms. Section 2.2 aims to equip AGRN with the capability to acquire structural chemical knowledge through the definition of molecular reconstruction losses. Lastly, Section 2.3 endeavors to enhance the success rate of discovering highly active ligands by meticulously designing the ligand activity optimization scheme and incorporating validity regularization techniques.

### 2.1 The attentive graph reconstruction neural network

**Figure 2.**
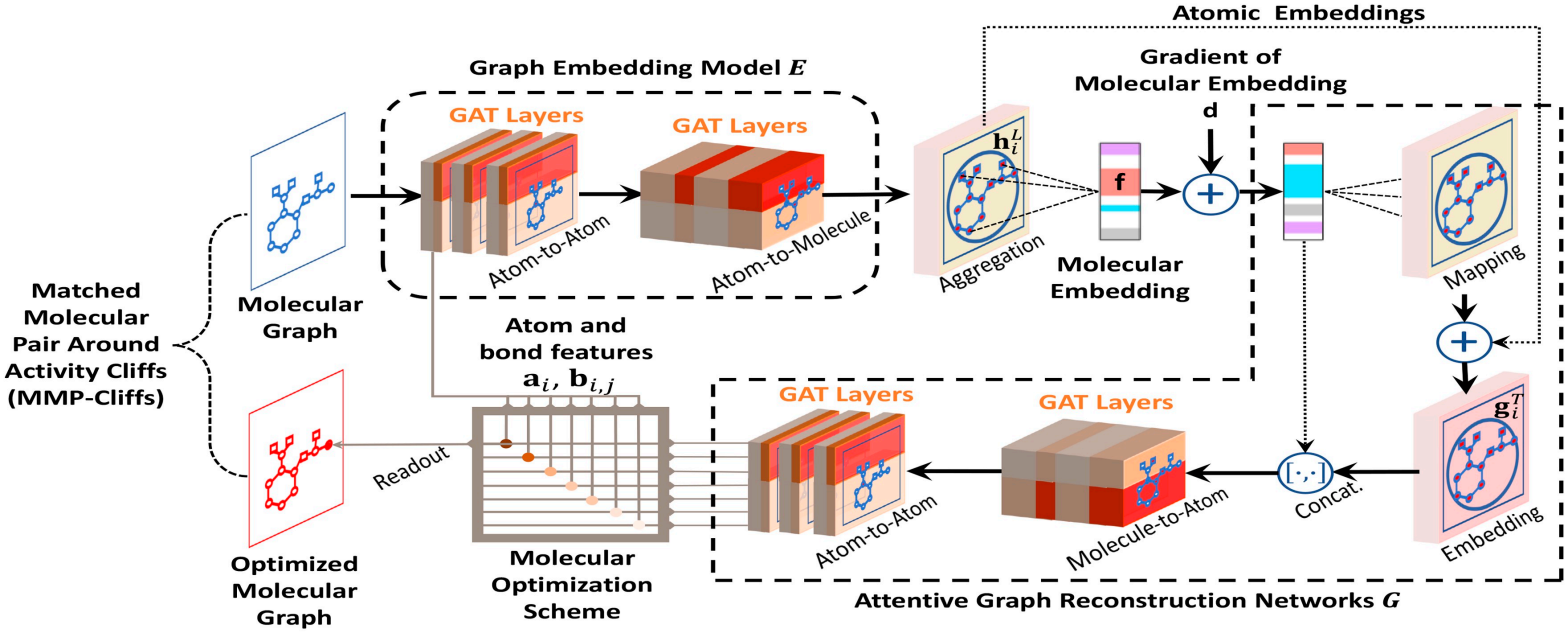
An illustration of ligand bioactivity optimization in the way of matched molecular pairs using OLB-AC.

The attention mechanism employed in graph representation has demonstrated its effectiveness and interpretability in extracting molecular embeddings, as validated in Xiong *et al.* (2019). Its capability to focus on essential atoms highlights its potential (Yin *et al.* 2021a). However, this alone is not sufficient for drug discovery as further modifications and optimizations of these key atoms are crucial for the development of molecular drugs. To address this, we present a novel AGRN as shown in Fig. 2. The AGRN reconstructs atomic and bond chemical features based on molecular and atomic embeddings, following five main steps.

#### 2.1.1 Molecular optimization clues

Yin *et al.* (2022a) introduced AFSE to improve the model generalization for ligand bioactivity prediction. They minimized the maximum prediction discrepancy on perturbed molecular features f+d in a feature subspace. The resulting adversarial perturbation **d** is used as clues for molecular optimization in this paper, which can be obtained by:

(1) $$L_{\text{AFSE}}(f,N,d):=D(N(f,f),N(f,f+d)),$$

(2) $$d=\eta \frac{g}{\left||g|\right|},g=\nabla_{r}D(N(f,f),N(f,f+r)){|}_{||r||\leq \varepsilon },$$

where *η* is the learning rate, ||·|| is the *l*_2_-norm, and *ε* is a small number. This process starts with a ligand embedding **f** extracted by DGL models, which will be fed into a neural network N for property prediction. In Equation (2), the feature vector **f** is perturbed with a random Gaussian vector **r** within the feature subspace. Then, the difference between the original predictions N(f,f) and the predictions after perturbation N(f,f+r) is computed by the measure D(·,·). Equation (1) further adjusts the feature vector **f** in the direction of the gradient of **r** to obtain an adversarial perturbation **d**. This perturbation indicates a significant change in model predictions and can help optimize ligands. Meanwhile, Equation (1) stabilizes model predictions on the perturbed feature f+d, enhancing the predictor N’s robustness for similar molecules with different properties. This improves model generalization, especially in the presence of ACs.

#### 2.1.2 Molecular embedding decomposition

In GNNs, the molecular embedding **f** is commonly obtained by aggregating the atomic embeddings $h_{i}^{L}$ for $i=1,2,\ldots ,N_{a}$, where *L* is the number of layers and *N_a_* is the number of atoms in the molecule. Aggregation methods such as summation are commonly used (Sun *et al.* 2020). If the atomic embedding $h_{i}^{L}$ has a smaller angle with the changed molecular embedding f+d, and a larger magnitude, the *i*-th atom is considered to be more responsible for the change. Therefore, we use the inner product between f+d and $h_{i}^{L}$ to quantify the atomic responsibility (denoted as *γ_i_*), and normalize these quantified values over all atoms in the molecule as follows:

(3) $$\gamma_{i}=\frac{\;\text{exp}(\langle f+d,h_{i}^{L}\rangle )}{\sum_{j}\;\text{exp}\;(\langle f+d,h_{j}^{L}\rangle )},$$

where 〈·〉 represents the vector inner product, and “exp” represents the exponential function. Then, $\gamma_{i}(f+d)$ serves as the component of the changed molecular embedding f+d on its *i*-th atom.

#### 2.1.3 Molecule-to-atom update

After decomposing the changed molecular embedding f+d into its atomic components $\gamma_{i}(f+d)$, the subsequent task is to estimate the new atomic embeddings $g_{i}$ that correspond to the changed molecular embedding f+d. To approach $g_{i}$ in one step, we implement a vector sum between the atomic components $\gamma_{i}(f+d)$ and the original atomic embeddings $h_{i}^{L}$:

(4) $$g_{i}^{T}=\gamma_{i}(f+d)+h_{i}^{L}.$$

Here, *T* is the number of steps to approach the new atomic embeddings $g_{i}$. To uncover the relationship information $r_{i}$ between the changed molecular embedding f+d and its corresponding atomic embeddings $g_{i}$, we employ a one-layer neural network (NN) and a gated recurrent unit (GRU). This process is iterated *T* times:

(5) $$r_{i}^{t}=\text{elu}(W\cdot (\text{dropout}([f+d,g_{i}^{t}]))+c),$$

(6) $$g_{i}^{t-1}=\text{relu}(\text{GRU}(r_{i}^{t},g_{i}^{t})).$$

In the update steps t=T,T−1,…,1 of Equation (6), the relationship information $r_{i}^{t}$ between the hidden embedding $g_{i}^{t}$ of each atom and the changed molecular embedding f+d is modeled using one-layer NNs and a GRU. The weight matrix **W** and bias vector **c** of the NNs are included, and the row concatenation is represented as [·,·]. To prevent overfitting, some network nodes are randomly dropped during training using dropout. The output of the neurons is activated nonlinearly using the Exponential Linear Unit (elu), which retains the nonlinear activation value of the negative part, while the Rectified Linear Unit (relu) is used for the output of nonlinearly activated neurons.

#### 2.1.4 Atom-to-atom update

To focus on neighboring atoms j∈N(i) that are critical to inferring the *i*-th atom’s chemical features, the attention weight *w_ji_* is learned with:

(7) $$w_{ji}=\text{softmax}(\text{leaky}\_\text{relu}(W\cdot \text{dropout}([g_{i}^{l},g_{ji}^{l}])+c)).$$

In Equation (7), the starting values of $g_{i}^{l=L}$ and $g_{ji}^{l=L}$ are taken from $g_{i}^{t=0}$ and $g_{j}^{t=0}$ in Equation (6). The activation function leaky relu is used, which allows a small slope for negative values in order to preserve information. To capture the chemical properties of atoms and bonds, the context information of each atom is aggregated, readout, and updated as follows:

(8) $$C_{i}^{l}=\text{elu}(\sum_{j}w_{ji}\cdot W(\text{dropout}(g_{ji}^{l}))+c),$$

(9) $$g_{i}^{l-1}=\text{relu}(\text{GRU}(C_{i}^{l},g_{i}^{l})),$$

(10) $$g_{ji}^{l-1}=\text{leaky}\_\text{relu}(W\cdot \text{dropout}([g_{i}^{l},g_{\text{ji}}^{l}])+c),.$$

The steps in Equation (10) are updated for l=L,L−1,…,1, where *L* is the same number of GNN layers as in the encoder.

#### 2.1.5 Ligand graph generation

To determine the chemical features of each atom and bond in ligands, the NNs and activation functions are utilized:

(11) $${\tilde{a}}_{i}=\phi_{a}(W\cdot g_{i}^{l=0}+c)$$

(12) $${\tilde{b}}_{i,j}=\phi_{b}(\text{leaky}\_\text{relu}(W\cdot \text{dropout}([g_{i}^{l=0},g_{ji}^{l=0}])+c)).$$

The functions $\phi_{a}$ and $\phi_{b}$ map the hidden embeddings to the chemical features of atoms and bonds, respectively. The definitions of the chemical features of atoms and bonds can be found in Supplementary Table S2.

Overall, the AGRN can be viewed as a graph decoder G that reconstructs and optimizes ligand graphs as follows:

(13) $$\text{Reconstruction:}\;\{f,\;H\}\to {\left\{{\hat{a}}_{i},\;{\hat{B}}_{i}\right\}}_{i=1}^{N_{a}},$$

(14) $$\;\text{where}\;H={\left\{h_{i}\right\}}_{i=1}^{N_{a}},\;{\hat{B}}_{i}={\left\{{\hat{b}}_{i,j}\right\}}_{i=1,\;j\in N(i)}^{i=N_{a},\;j\in N(i)},$$

(15) $$\text{Optimization:}\;\{f+d,\;H\}\to {\left\{{\tilde{a}}_{i},\;{\tilde{B}}_{i}\right\}}_{i=1}^{N_{a}},$$

(16) $$\;\text{where}\;H={\left\{h_{i}\right\}}_{i=1}^{N_{a}},\;{\tilde{B}}_{i}={\left\{{\tilde{b}}_{i,j}\right\}}_{i=1,\;j\in N(i)}^{i=N_{a},\;j\in N(i)},$$

where *N_a_* represents the number of atoms in the molecule, and *N*(*i*) is the index set of adjacent atoms of the *i*-th atom. The AGRN algorithm is detailed in Supplementary Algorithm S2.

### 2.2 Ligand graph reconstruction

To facilitate AGRN to acquire structural chemistry knowledge, we design a reconstruction loss function that allows AGRN to learn from reconstructing ligand graphs:

(17) $$L_{\mathrm{Recon}.}({\hat{a}}_{i},a_{i},{\hat{b}}_{i,j},b_{i,j}):=$$

(18) $$\frac{1}{N_{a}}\sum_{i=1}^{N_{a}}[L_{a}({\hat{a}}_{i},a_{i})+\sum_{j\in N(i)}L_{b}({\hat{b}}_{i,j},b_{i,j})].$$

The reconstruction error functions, *L_a_* and *L_b_*, are designed to minimize the error between the reconstructed chemical features (${\hat{a}}_{i},\;{\hat{b}}_{i,j}$) and the actual chemical features ($a_{i},\;b_{i,j}$), as shown in Supplementary Table S2. The adjacent atom index set *N*(*i*) contains the index of the atom adjacent to the *i*-th atom.

Natural molecules contain varying amounts of different elements, resulting in a prior distribution of chemical elements. To account for this variation, the elements are weighted according to their proportions in each molecule when calculating the cross-entropy loss of node classification, denoted as “WCE” in Supplementary Table S2. The original atomic symbol $a_{i,k=K_{i}}=1$ if the *i*-th atom belongs to the *k*-th element, while the reconstruction probability ${\hat{a}}_{i,k}\in [0,1]$ measures the probability that the *i*-th atom belongs to the *k*-th element. The weighted cross-entropy loss is defined as shown in Equation (19):

(19) $$\text{WCE}(\hat{a},a):=-\sum_{i=1}^{N_{a}}\left(1-\frac{N_{k=K_{i}}}{N_{a}}\right)\cdot \text{CE}({\hat{a}}_{i},a_{i}),$$

where $N_{k}=\sum_{i=1}^{N_{a}}a_{i,k}$, and *K_i_* denotes the index of the original symbol of atom $a_{i}$. “CE” refers to cross-entropy loss.

### 2.3 Ligand graph optimization

#### 2.3.1 Ligand bioactivity optimization scheme

Refining ligands chemically to enhance their bioactivity leads to the emergence of matched molecular pairs near the AC (referred to as MMP-Cliffs) (Cruz-Monteagudo *et al.* 2014), which hold significant importance in the exploration of ACs and ligand bioactivity optimization. The basic chemical transformation involves modifying an element at an atomic site. In drug lead discovery, the binding sites of drug molecules to targets are often located at key atomic sites, which typically have distinct topological relationships with other atomic sites (Zhao *et al.* 2020). Therefore, the ligand bioactivity optimization in this paper changes the element symbols of one atom at a time by using a graph node optimization scheme, resulting in the creation of MMP-Cliffs. This is illustrated in Fig. 2, which also facilitates the interpretation of gradients in molecular embeddings.

The critical embedding f+d of changing model predictions (introduced in Section 2.3.1) can help identify a key atomic site for replacing elements. With the embedding f+d, the key atomic positions are identified by determining the distribution of posterior probabilities. Let P(k|i) be the posterior probability that the *i*-th atom is predicted to be the *k*-th chemical element, which can be estimated by AGRN:

(20) $$\text{Reconstruction:}\;P_{f}(k|i):={\hat{a}}_{i,k},$$

(21) $$\text{Optimization:}\;P_{f+d}(k|i):={\tilde{a}}_{i,k},$$

(22) $$\text{where}\;i=1,2,\ldots ,N_{a},\;k=1,2,\ldots ,N_{s}.$$

The variables ${\hat{a}}_{i,k}$ and ${\tilde{a}}_{i,k}$ represent the probability estimates of assigning the *k*-th chemical element to the *i*-th atom, as reconstructed and optimized by AGRN. The variable *N_a_* represents the total number of atoms in the ligand. The number of common chemical elements *N_s_* is 16, as indicated by the description of atomic symbols in Supplementary Table S2. The key atomic position $i^{*}$ is then identified based on the maximum posterior probability criterion as follows:

(23) $$i^{*}=\underset{i}{\arg\max}\left(\underset{k\notin K_{i}}{\max}P_{f+d}(k|i)\right),$$

(24) $$\text{where}\;K_{i}:=\{k|P_{f}(k|i)\geq P_{0}\}.$$

In Equation (24), the chemical elements within the set $K_{i}$ exhibit a reconstruction confidence $P_{f}(k|i)$ that surpasses the threshold *P*_0_. These elements correspond to either the original or potentially misleading chemical elements of the *i*-th atom, as discussed in Yin *et al.* (2021b, 2021c, 2022b). To generate true novel chemical elements for the *i*-th atom, we need to ensure that $k\notin K_{i}$. The final key atom position $i^{*}$ is determined by having the maximum optimization confidence $P_{f+d}(k|i)$ among all atoms and chemical elements *k* not in $K_{i}$. The replacement element $k^{*}$ is then determined based on the maximum posterior probability as follows:

(25) $$k^{*}=\underset{k\in K_{i}^{*}}{\arg\max}P_{f+d}(k|i^{*}),$$

(26) $$\text{where}\;K_{i}^{*}:=\{k|P_{f+d}(k|i^{*})\geq P_{0},k\neq K_{i^{*}}\}.$$

**Figure 3.**
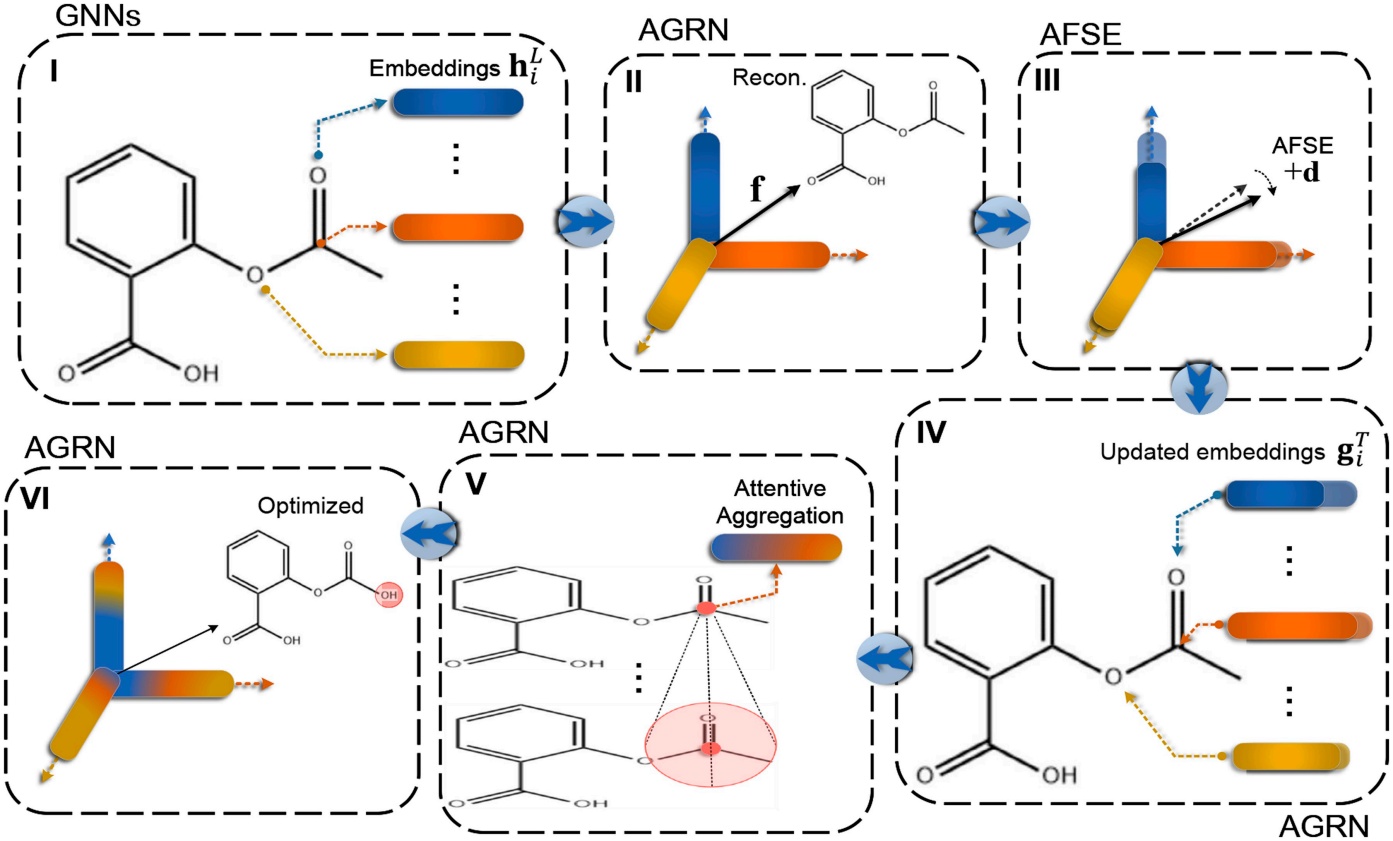
A schematic of ligand bioactivity optimization in OLB-AC: (I) Graph embeddings $h_{i}^{L}$ for atoms. (II) Aggregating into the molecular embedding **f** and reconstructing the molecular graph. (III) Mapping of the **d** generated by AFSE onto atom embeddings. (IV) Updating of atom embeddings $g_{i}^{T}$. (V) Graph attention aggregation among atom embeddings. (VI) Selecting the atom and the replaced element based on updated atom embeddings.

In Equation (26), the chemical elements in the candidate set $K_{i}^{*}$ are those with an optimization confidence $P_{f+d}(k|i^{*})$ greater than *P*_0_ and that differ from the original chemical element $K_{i^{*}}$ of the key atom position $i^{*}$. This eliminates the effect of low-confidence optimization. The diagram of the OLB-AC ligand bioactivity optimization is illustrated in Fig. 3, and its implementation can be found in Supplementary Algorithm S3.

#### 2.3.2 Ligands validity optimization

The validity of the elements in a ligand must comply with chemical regulations. To ensure this, this paper proposes a novel mask-based objective for ligand validity optimization:

(27) $$L_{\mathrm{Val}.}(i^{*},k^{*}):=-\frac{1}{N_{a}}\sum_{i=1}^{N_{a}}\text{Val}(k^{*}|i^{*})\cdot \;\text{log}(1-P_{f+d}(k^{*}|i^{*})),$$

(28) $$\text{where Val}(k^{*}|i^{*})=\{\begin{array}{c} 1,\;\text{invalid assignment}\\ 0,\;\text{otherwise}. \end{array}$$

In Equation (28), invalid ligands are filtered using the mask function $\text{Val}(k^{*}|i^{*})$, and a penalty term is imposed on their violation probabilities $P_{f+d}(k^{*}|i^{*})$.

#### 2.3.3 Total optimization objective

Overall, OLB-AC integrates four optimization objectives: (1) biological objective $L_{\mathrm{Bio}.}$, (2) AFSE objective $L_{\mathrm{AFSE}}$, (3) reconstruction objective $L_{\mathrm{Recon}.}$, and (4) validity objective $L_{\mathrm{Val}.}$. The overall optimization objectives can be expressed as follows:

(29) $$\underset{E,N,G}{\min}\underset{d}{\max}\;\underset{\text{Representation}\;\text{Learning}}{\underset{︸}{L_{\mathrm{Bio}.}+\lambda_{1}L_{\mathrm{AFSE}}}}+\lambda_{2}\underset{\text{Ligand}\;\text{Optimization}}{\underset{︸}{\left(L_{\mathrm{Recon}.}+L_{\mathrm{Val}.}\right)}},$$

(30) $$\text{where}\;L_{\mathrm{Bio}.}:=L(N(f,f),y),$$

The label *y* denotes the experimentally determined ligand bioactivity or property value through chemical wet experiments. The error function, L(·,y), represents the difference between predictions and the true assay values and can be expressed as a cross-entropy loss for classification assays or a mean square error for regression assays. The coefficients *λ*_1_ and *λ*_2_ are used to balance the biological loss and the AFSE loss, as well as the representation learning and ligand bioactivity optimization objectives, respectively.

## 3 Experiments

### 3.1 Test of OLB-AC on ligand bioactivity and property modeling

OLB-AC optimizes ligand molecules based on adversarial gradients during ligand bioactivity and property prediction. Therefore, precise prediction on them is fundamental for the optimization, and this section assesses the effectiveness of OLB-AC in this regard.

#### 3.1.1 Datasets

This section involves two publicly available benchmark datasets from AFSE Yin *et al.* (2022a) (temporarily denoted as Activity-33 in this study) and ADMETlab2.0 Xiong *et al.* (2021) (temporarily denoted as **A**DMET-25 in this study). The Activity-33 is the benchmark for the modeling of bioactivities on ligand molecules, which contains 33 datasets constructed by Yin *et al.* (2022a). There are 33 representative GPCR bioactivity datasets downloaded from the GLASS database (Chan *et al.* 2015) with various data sizes. Specifically, there are a considerable amount of inactive ligands in their test sets to evaluate the model generalization ability on real data distributions (Yin *et al.* 2021a). In Activity-33, receptor IDs are taken from the UniProt database.

The ADMET-25 benchmark contains 25 publicly available datasets on drug absorption, distribution, metabolism, and toxicity provided by ADMETlab2.0 Xiong *et al.* (2021). Their data sources included open-access bioactivity databases, such as ChEMBL (Mendez *et al.* 2019), PubChem (Kim *et al.* 2021) and OCHEM (Sushko *et al.* 2011), peer-reviewed literature, and freely accessible software Toxicity Estimation Software Tools (TEST) developed by the U.S. Environmental Protection Agency (EPA 2001).

#### 3.1.2 Performance indexes

To assess the model performance in the benchmark of ligand bioactivity prediction (Activity-33), we employ two typical regression performance indexes: the square of Pearson correlation coefficient (*r*^2^) and the root mean square error (RMSE). The larger the *r*^2^ or the lower the RMSE, the higher the accuracy of the bioactivity prediction.

To assess the model performance in the benchmark of ligand property prediction (ADMET-25), we employ five classifications performance Indexes, including the area under the receiver operating characteristic curve, accuracy (ACC), the Matthews correlation coefficient, specificity, and sensitivity. The larger these indexes are, the more accurate the MP is.

The definitions of the above performance indexes are detailed in Supplementary Text S1.

#### 3.1.3 Baselines

In the benchmark of Activity-33, we compared OLB-AC with other DGL models as well as basic QSAR models. The basic QSAR models include simple random forest (RF) and multi-layer perceptron (MLP) based on Morgan fingerprints. The DGL models involve graph attention networks (GATs) (Veličković *et al.* 2017), graph isomorphism networks (GINs) (Xu *et al.* 2018) pre-trained with supervised learning and context prediction (Hu *et al.* 2019), neural fingerprint (Neural FP) (Duvenaud *et al.* 2015), Weave (Kearnes *et al.* 2016), message passing neural networks (MPNNs) (Gilmer *et al.* 2017). We also compared OLB-AC with the backbone model, Attentive FP and AFSE, and the state-of-the-art model Uni-Mol. Attentive FP is a graph NN architecture for molecular representation that uses a graph attention mechanism to learn from relevant drug discovery datasets (Xiong *et al.* 2019). AFSE is an algorithm that dynamically generates critical representations in a new feature subspace via bi-directional adversarial learning. It then minimizes the maximum loss of prediction divergence on bioactivity to ensure the local smoothness of model outputs (Yin *et al.* 2022a). Uni-Mol is a universal 3D molecular representation learning framework that significantly enlarges the representation ability and application scope of molecular representation learning schemes (Zhou *et al.* 2023).

In the benchmark of ADMET-25, we compared OLB-AC with the benchmarked method ADMETlab2.0 (Xiong *et al.* 2021). ADMETlab 2.0 is an enhanced version of the widely used ADMETlab (Dong *et al.* 2018) for systematic evaluation of ADMET properties, as well as some physicochemical properties and medicinal chemistry friendliness.

#### 3.1.4 Settings

To improve training efficiency, we focused on the molecular reconstruction of atomic symbols that require optimization. The architecture of AGRN includes the same number of GNN layers as the graph encoder, with *T *=* *1 and *L *=* *2. The balance coefficients $\lambda_{1}=0.6$ and $\lambda_{2}=0.3$ are employed, and a learning rate schedule is used to warm up and cool down model learning. The model study with different hyperparameters can be found in Section 3.4.

#### 3.1.5 Results

As shown in Table 1, Supplementary Tables S8 and S9, OLB-AC outperforms other methods on the majority of datasets (27/33 on *r*^2^ and 26/33 on RMSE) and achieves the highest overall performance, with improvements ranging from 4.25% to 22.93% over state-of-the-art methods. When compared to AFSE, OLB-AC exhibits an average improvement of 5.72% across all 33 tasks, and an average improvement of 13.85% when compared to Attentive FP. The results reveal that basic QSAR methods (RF and MLP) utilizing Morgan fingerprints can outperform certain DGL methods when ACs are present, consistent with the conclusion drawn in Dablander *et al.* 2023. In Table 1, OLB-AC demonstrates consistent performance across the typical task set, with performance fluctuations on five random initializations not exceeding 8.25%, and an average fluctuation of 4.27%. These results underscore the superior generalization performance of OLB-AC, which integrates molecular graph reconstruction and optimization. This suggests that while the model learns to optimize the ligand’s bioactivity, it also enhances its comprehension of the ligand molecule’s structure–activity relationship.

**Table 1.** Comparison of the square of Pearson correlation coefficient *r*^2^ index on the ligand bioactivity prediction benchmark (A typical subset in Activity-33).^a^

Methods		Fingerprint-based		Graph-based								
Dataset size	Task ID^b^	RF	MLP	GATs	GINs	Neural FP	Weave	MPNN	Attentive FP	Uni-Mol	AFSE^c^	OLB-AC^d^
Small (200–400)	1	0.0031	−0.0812	0.1576	0.1347	0.0384	0.2785	0.1551	0.2508	0.2983	0.4347	0.4553 ± 0.0212
	4	0.4473	0.1627	0.1207	0.0645	0.0032	0.1132	0.0079	0.2786	0.3995	0.4660	0.4770 ± 0.0192
	7	0.2071	0.1775	0.2871	0.0360	0.0437	0.2871	0.0008	0.1866	0.2770	0.5079	0.5755 ± 0.0351
Medium (1000–2000)	10	0.4958	0.4981	0.4086	0.3585	0.0223	0.5076	0.5086	0.4764	0.4356	0.5102	0.5458 ± 0.0194
	13	0.2183	0.2098	0.2215	0.1274	0.0008	0.2760	0.3014	0.3690	0.3297	0.3702	0.3984 ± 0.0189
	17	0.1485	0.1284	0.1870	0.0364	0.1044	0.2325	0.2571	0.2400	0.2079	0.3032	0.3143 ± 0.0259
Large (2000–4000)	21	0.3458	0.4238	0.5636	0.4297	0.0779	0.4964	0.4459	0.5164	0.4333	0.5173	0.5783 ± 0.0235
	28	0.2662	0.2973	0.3673	0.1815	0.0095	0.3270	0.4229	0.3782	0.2870	0.4087	0.4619 ± 0.0056
	33	0.3402	0.5144	0.1259	0.1947	0.0214	0.5589	0.5171	0.5088	0.5090	0.5025	0.5551 ± 0.0177
Average		0.2747	0.2590	0.2710	0.1737	0.0357	0.3419	0.2908	0.3561	0.3530	0.4467	0.4846 ± 0.0207

a Baseline results are taken from Yin *et al.* (2022a). More results can be found in Supplementary Tables S8 and S9.

b Under the same dataset size (the ranges of the total number of molecules in each kind of dataset size are shown in brackets), the larger the value of Task ID, the greater the difference in bioactivity distribution between test and training data.

c Here, AFSE takes the Attentive FP as the backbone network.

d The mean and standard deviation of the accuracy were recorded over five random initializations.

Supplementary Table S10 illustrates that the OLB-AC method outperforms ADMETlab 2.0 in the majority of performance indexes for all 25 ADMET classification tasks. On average, the area under the precision–recall curve is improved by 5.03%, the classification accuracy (ACC) by 3.64%, the Matthews correlation coefficient by 29.07%, the specificity of binary classification by 4.23%, and the sensitivity of binary classification increased by 5.90% when using OLB-AC. These results reveal the ability of OLB-AC as an accurate property predictor to provide clues for its molecular optimization.

### 3.2 Optimizing ligand bioactivities through OLB-AC

This section verifies OLB-AC’s ability to optimize ligands toward a higher activity that matches existing ligands. By default, OLB-AC employs the perturbed features f+d to optimize molecules, aiming to enhance their activity. However, an intriguing question arises: Can OLB-AC utilize the features of reverse perturbation, f−d, to reverse optimize the molecule, thereby reducing its activity? This section also investigates this aspect within the framework of reverse optimization settings.

#### 3.2.1 Datasets and settings

This section encompasses three typical tasks (Task 12: P21453, Task 19: O43614, and Task 31: P14416) within the Activity-33 datasets, each characterized by a relatively higher abundance of MMP-Cliffs. These MMP-Cliffs denote ligands that differ by only one atom but exhibit bioactivity values differing by more than 10 times, categorized as highly active ligands and low-activity ligands.

1. In the optimization experiment, we screened for MMP-Cliffs and used the remaining ligands as the training set. We then put the low-activity ligands in MMP-Cliffs into the training set to see if the OLB-AC-optimized ligands include the ones with higher activity values in MMP-Cliffs.
2. In the reverse optimization experiment, the high-activity molecules on MMP-Cliffs are included in the training set, while the corresponding low-activity molecules are exclusively placed in the test set.

#### 3.2.2 Performance indexes

We evaluate the optimization performance using four performance indexes, including the number of generated molecules and discovered high-activity molecules, as well as the maximum and average predicted bioactivity on the generated molecules by well-trained models in the bioactivity prediction experiments. Additionally, for individual molecules, we evaluate the ligand’ bioactivity to targets (Activity, measured by pIC50, pEC50, pKi, or pKd) and three typical molecular properties, including quantitative estimation of drug-likeness, synthesizability, and oil–water partition coefficient (LogP). Quantitative estimation of drug-likeness ranges from 0 to 1, with higher values indicating higher drug-like properties. Synthesizability ranges from 1 to 10, with closer values to 1 indicating easier synthesis. Drugs are well absorbed when LogP values are between 0 and 3. Values outside of this range may hinder drug absorption.

#### 3.2.3 Baselines

This section presents a comparative analysis of OLB-AC with three typical molecular optimization methods: a traditional MMP analysis method (MMPDB: Dalke *et al.* 2018), a de novo generative method (HN-GFN: Zhu *et al.* 2024), an MMP-based deep-learning method [temporarily referred to as “MMP-Optimizer” (He *et al.* 2021a) in this study]. For MMPDB, we iterate over all single transformations (which form MMPs) it identifies in the training set and apply them to the test molecules (if applicable). MMPDB predicts single transformations that result in increased bioactivity as its optimization results, whereas single transformations predicted to decrease bioactivity are considered its reverse optimization results. For HN-GFN and MMP-Optimizer, we utilize the codes and default hyperparameters available in their respective GitHub repositories^1,2^ on our datasets. In addition, we also randomly sample (referred to as “Random sampling”) 10 atoms for modification, and 10 chemical symbols are randomly chosen for each atom to replace the original chemical symbol.

#### 3.2.4 Results

**Figure 4.**
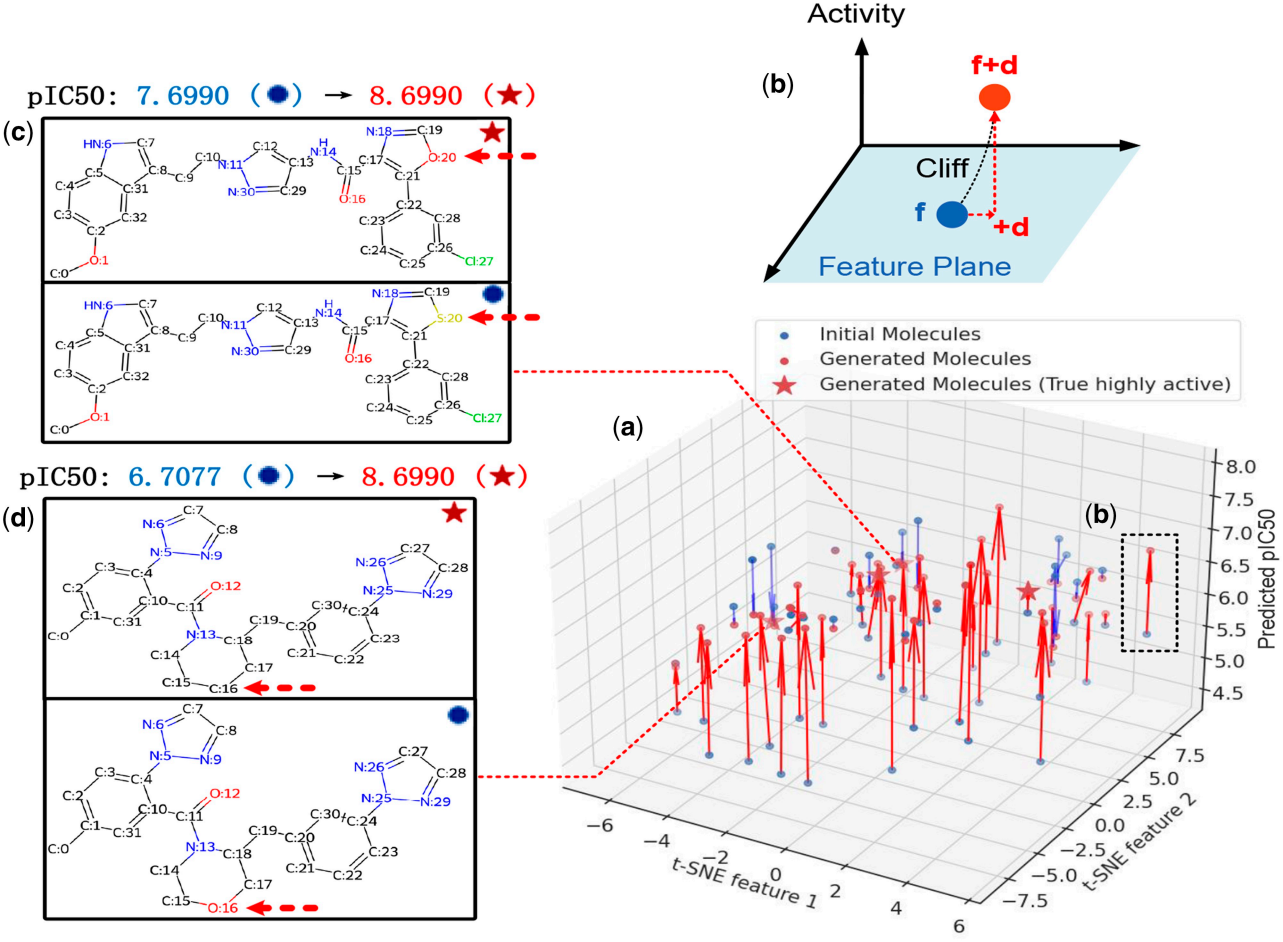
Visualization of OLB-AC ligand bioactivity optimization, with the example of the orexins receptor O43614: (a) The figure depicts the positioning of OLB-AC-optimized ligands in the predicted structural feature-activity space. The *x*–*y* plane illustrates the t-distributed stochastic neighbor embedding (t-SNE: Van der Maaten and Hinton 2008) of molecular graph features. The majority of OLB-AC-optimized molecules exhibit higher predicted pIC50 values than their initial molecules, as indicated by the red arrows. Some of these optimized molecules correspond to true highly active ligands for O43614. (b) An illustration showcases the OLB-AC optimization (+d) process on the individual ligand feature **f**. (c and d) Examples highlight true highly active ligands (denoted by stars), with red arrows indicating optimized atoms, obtained through OLB-AC optimization. Molecules marked with circles in the upper right corner represent the initial molecules utilized for optimization.

As an example, Fig. 4 illustrates the location of OLB-AC-optimized ligands in the predicted structural feature-activity space using the orexin receptor O43614 from the database (Task 19 in Activity-33). The results indicate that the majority of the optimized ligands have significantly increased predicted activities, and their structural features are similar to their original counterparts, fulfilling the criteria of MMP-Cliffs. In Fig. 4a, the projection of solid arrows on the x-y plane indicates OLB-AC’s **d**, which contains crucial information in optimizing ligands. An activity assay query on orexin receptor O43614 revealed that four optimized ligands (represented by red stars in Fig. 4a) have much higher true bioactivities compared to their original counterparts. Furthermore, the results of OLB-AC’s ligand bioactivity optimization can provide valuable insights to chemists by analyzing the changes in elements at single atomic positions between the optimized and original ligands (indicated by dotted arrows in Fig. 4b–e).

Table 2 provides a quantitative comparison of the performance of each method in molecular optimization. OLB-AC stands out by generating the fewest molecules yet discovering the most highly active molecules on the anchor, both in optimization and reverse optimization settings, compared to baseline methods. Our experiments reveal that the de novo molecule generation model (HN-GFN) and the MMP-based methods (MMPDB and MMP-Optimizer) often failed to discover highly active molecules among the limited generated molecules. Additionally, through random modification of a single atom element 30–33 times on 98–177 anchor molecules, we observed the discovery of highly active molecules in a few instances (with an empirical probability ranging from 0.018% to 0.03%). In the 17 highly active molecules optimized by OLB-AC, 5 revealed novel transformations not found in their training sets, which we listed in Table 3 and Supplementary Table S3.

**Table 2.** Quantitative comparison of molecular optimization performance in typical tasks of Activity-33.

Performance indexes	Settings	Optimization (↑)			Reverse optimization (↓)		
	Targets	P21453	O43614	P14416	P21453	O43614	P14416
Number of molecules	Anchor	98	81	177	98	81	177
Generated	Random sampling	3270	2680	5429	3250	2711	5363
	MMPDB	34 257	23 525	58 393	39 500	27 886	55 041
	HN-GFN	741	2726	1693	14 131	10 712	14 623
	MMP-Optimizer	970	800	1750	980	810	1750
	OLB-AC	**84**	**81**	**76**	**80**	**57**	**140**
Discovered	Random sampling	1	0	1	0	0	0
	MMPDB	0	0	0	0	0	0
	HN-GFN	0	0	0	0	0	0
	MMP-Optimizer	0	0	0	0	0	0
	OLB-AC	**3**	**9**	**5**	**2**	**3**	**3**
Predicted bioactivity							
Max	Original	9.40	8.00	8.12	10.22	9.00	11.52
	Random sampling	9.73	8.28	7.89	10.36	8.60	9.10
	MMPDB	**10.65**	8.34	7.98	10.72	8.90	9.10
	HN-GFN	10.16	8.03	8.46	9.40	7.96	8.41
	MMP-Optimizer	9.82	8.16	8.38	8.63	8.03	**8.20**
	OLB-AC	10.11	**8.70**	**9.31**	**8.66**	**7.98**	8.19
Mean	Original	6.75	6.30	5.75	7.85	7.71	7.32
	Random sampling	5.28	5.75	6.20	6.48	5.94	5.80
	MMPDB	7.02	**6.81**	6.29	7.84	6.98	6.59
	HN-GFN	5.67	5.74	5.56	**3.16**	**1.92**	**2.73**
	MMP-Optimizer	7.08	6.21	6.43	6.62	6.62	6.48
	OLB-AC	**7.51**	6.64	**6.57**	6.63	6.93	6.53
	Measurement	pEC50	pIC50	pKi	pEC50	pIC50	pKi

The best performance in each index is bolded.

**Table 3.** The drug targets, common parts, novel transformations (not found in their training sets), and properties between original and OLB-AC optimized molecules.^a^

Targets	Common parts	Original properties	Novel transformations	Optimized properties
Human sphingosine 1-phosphate receptor (P21453)	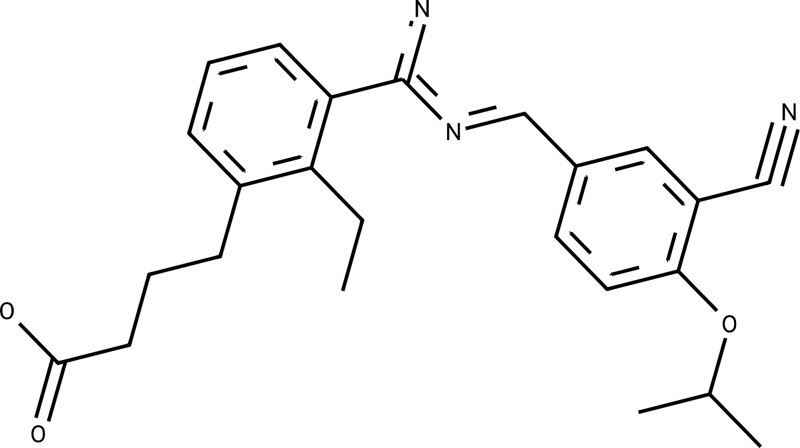	Activity^b^: 8.6002	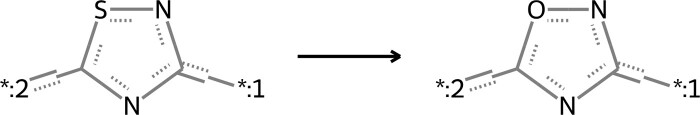	Activity: 9.6995 (++)
		QED^c^: 0.4766		QED: 0.5177 (+)
		SA^d^: 2.7684		SA: 2.5739 (+)
		logP^e^: 5.5007		logP: 5.0322 (+)
Human orexin type 2 receptor (O43614)	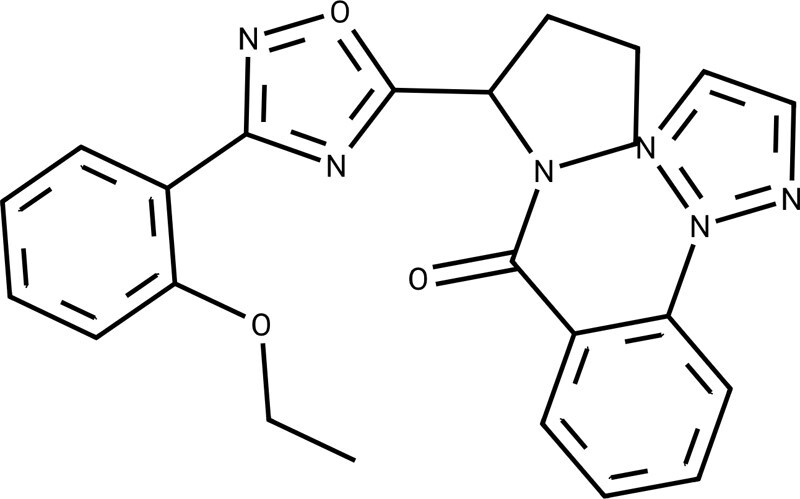	Activity: 6.9788	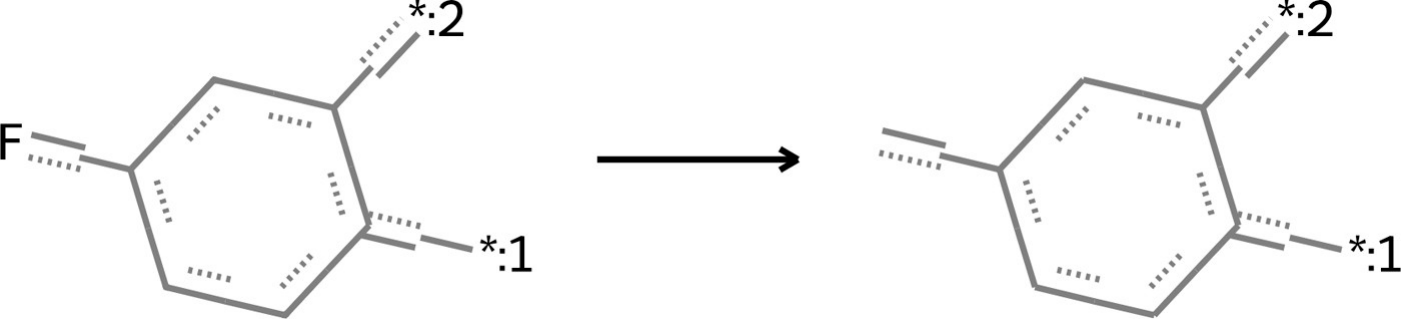	Activity: 8.3979 (++)
		QED: 0.4419		QED: 0.4431 (+)
		SA: 3.0856		SA: 3.0689 (+)
		logP: 3.8324		logP: 4.0017 (–)
	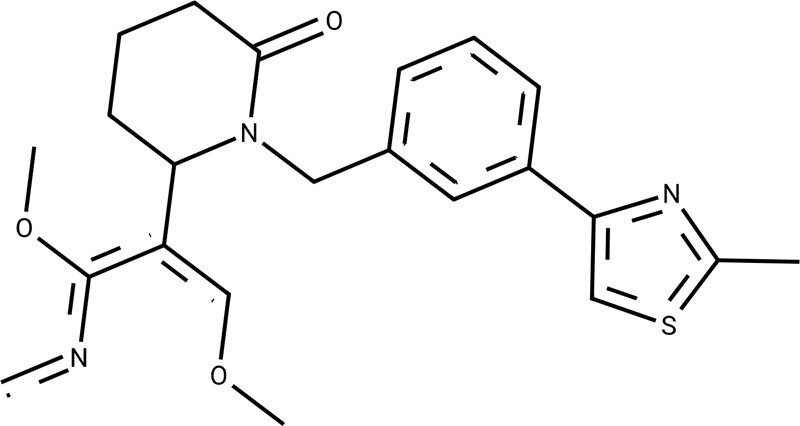	Activity: 6.2083	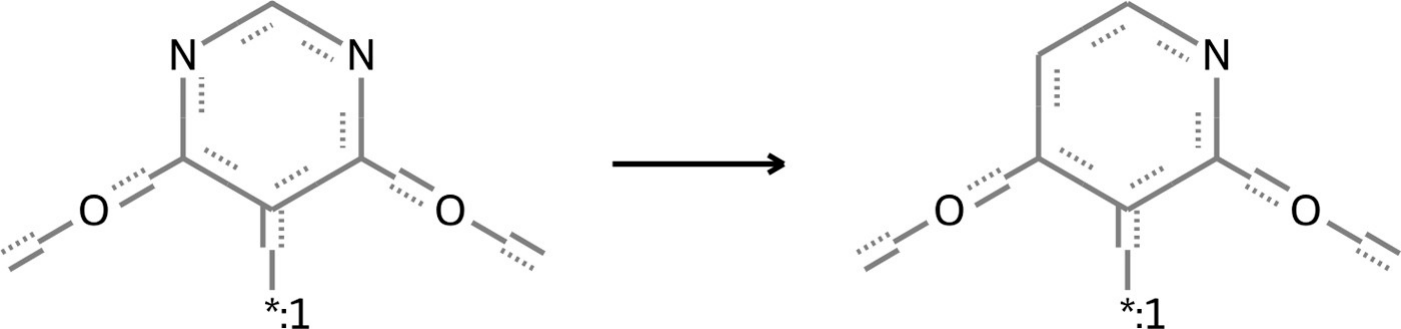	Activity: 7.8861 (++)
		QED: 0.5908		QED: 0.5706 (–)
		SA: 3.1402		SA: 3.0246 (+)
		logP: 4.1795		logP: 4.7845 (–)

a More results can be found in Supplementary Tables S3–S7.

b Activity: ligands’ bioactivity to targets, measured by pIC50, pEC50, pKi, or pKd.

c QED ranges from 0 to 1, with higher values indicating higher drug-like properties.

d SA ranges from 1 to 10, with closer values to 1 indicating easier synthesis.

e LogP: oil–water partition coefficient. Drugs are well absorbed when LogP values are between 0 and 3.

QED, quantitative estimation of drug-likeness; SA, synthesizability.

**Figure 5.**
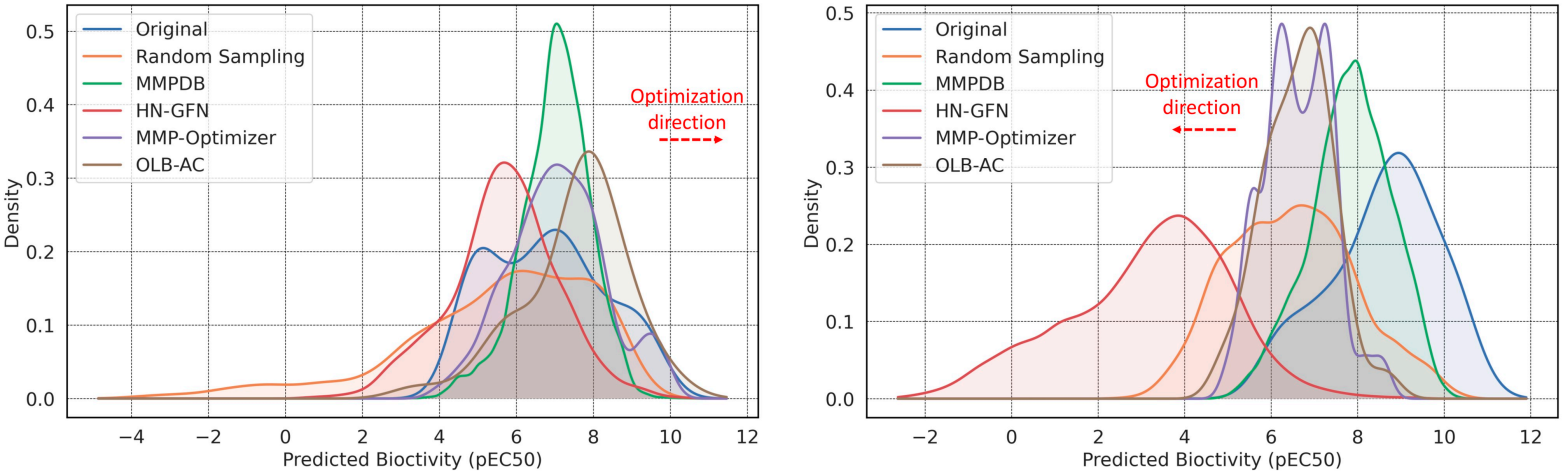
The predicted bioactivity distributions (left) between optimized molecules and the original low-activity molecules, and (right) between reverse-optimized molecules and the original high-activity molecules for the target P21453.

Furthermore, in conjunction with Fig. 5, we examined the distribution of predicted bioactivity values of the original molecules optimized through different methods. Notably, molecules optimized by OLB-AC exhibited higher predicted bioactivity values, with a distribution skewed toward the rightmost end in Fig. 5 (left). MMP-Optimizer followed, with the average predicted bioactivity after optimization being 6.07% lower than that of OLB-AC.

In reverse optimization experiments, MMP-based methods (MMPDB, MMP-Optimizer, and OLB-AC) performed worse than HN-GFN, which generates a large number of molecules from scratch with extremely low predicted bioactivity, without MMP constraints. Besides this, the predicted bioactivity distribution of molecules generated by OLB-AC in reverse optimization experiments closely resembles that of MMP-Optimizer, while molecules generated by random sampling exhibit a wider predicted bioactivity range.

In Supplementary Table S4, we showcase representative cases of high-activity ligands optimized by OLB-AC, including Task 12 (P21453), Task 19 (O43614) and Task 31 (P144161) in the Activity-33 benchmark. “Anchor Ligands” refer to low-activity ligands in the training set, and “Optimized Ligands” are the high-activity ligands produced by OLB-AC based on these “Anchors.” The results obtained from various datasets highlight the capability of OLB-AC in generating highly active ligands through the modification of a single atom symbol while maintaining acceptable pharmaceutical properties.

### 3.3 Enhancing inhibitory efficacy and reducing toxicity through OLB-AC’s ligand bioactivity optimization

In drug discovery, modeling ligand bioactivity is crucial for discovering drug leads. In this paper, ligand molecules are represented as graphs and analyzed using DGL. This approach can be extended to other tasks in drug lead discovery, such as molecular property optimization. Therefore, we extend OLB-AC to enhance ligand inhibitory efficacy and reduce ligand toxicity. This extension aims to aid in the understanding of molecular property changes and provide a reference for molecular property optimization. We present the numbers of anchors, generated, discovered molecules, and novel transformations in Table 4. Further details can be found in Supplementary Text S5–S7.

**Table 4.** The numbers of anchors, OLB-AC generated, discovered molecules and novel transformations in the representative tasks of ADMET-25.

Property	Inhibitor			Toxicity		
Targets	CYP1A2	CYP2C9	CYP3A4	NR-AhR	NR-ER	AMES
# Anchor^a^	30	73	51	67	85	312
# Generated	27	31	34	46	69	219
# Discovered^b^	2	2	2	5	5	16
# Novel Trans.^c^	2	2	1	5	1	11
Success rate^d^	7.41%	6.45%	5.88%	10.87%	7.25%	7.31%
Novel rate^e^	100.00%	100.00%	50.00%	100.00%	20.00%	68.75%

a Noninhibitor or toxic ligands for the target.

b Inhibitor or nontoxic ligands for the target.

c Among discovered molecules, the number of novel transformations that are not found in their training sets.

d “# Discovered” divided by “# Generated.”

e “# Novel Trans.” divided by “# Discovered.”

### 3.4 Model study on OLB-AC

This section investigates the model parameters, training time, loss functions, hyperparameters, and training process on OLB-AC in Task 7 of Activity-33.

1. **Model parameters and training time:** We conducted a comparison of the model parameters and training time of OLB-AC with baseline methods. As depicted in Supplementary Table S11, the model parameter size of OLB-AC is 520KB, representing a moderate model size compared to baseline methods. The training time for OLB-AC was recorded at 18.67 minutes. Despite having the longest training time among baseline methods (8.47 minutes longer than the second one, i.e., AFSE), OLB-AC achieved the highest prediction performance and was capable of simultaneously optimizing ligand molecules. In summary, OLB-AC utilizes approximately 53% of the model parameters and 45% of the training time compared to its predecessor (AFSE) for additional ligand molecule reconstruction and optimization.
2. **Loss functions:** We explored the impact of reconstruction loss and validity loss on OLB-AC molecular optimization. As illustrated in Supplementary Fig. S1, compared to the original molecule (denoted as “original”), molecules optimized by OLB-AC exhibit higher predicted bioactivity values (shifted toward the right). Within OLB-AC, molecules optimized with all losses (denoted as “optimized”) yield the highest predicted bioactivity value compared to scenarios where reconstruction loss (denoted as “optimized w/o recon”) or validity loss (denoted as “optimized w/o valid”) are not utilized. This finding underscores the essential role of valid molecule reconstruction in OLB-AC molecular optimization. Moreover, reconstruction loss plays a more crucial role than validity loss in the optimization process.
3. **Hyperparameters:** We conducted tests with OLB-AC using different values of the aggregation step [*T* in Equation (4)] and the balance coefficients of the loss function [*λ*_1_ and *λ*_2_ in Equation (30)] to assess performance. In Supplementary Fig. S2(a), we observe that increasing *T* values from 1 to 4 initially leads to a decrease in OLB-AC performance, followed by an improvement to a comparable level. Further increasing *T* to 5 results in additional performance enhancement. However, considering training cost, we opt for *T* = 1 due to its relatively high performance-to-cost ratio.
  In Supplementary Fig. S2(b), we incrementally increase the values of *λ*_1_ and *λ*_2_ from 0 to 1 in steps of 0.1. The results indicate that OLB-AC achieves optimal performance when $\lambda_{1}=0.6$ and $\lambda_{2}=0.3$. Additionally, when varying the values of *λ*_1_ and *λ*_2_ between 0 and 1 in OLB-AC, the change in performance on test *r*^2^ does not exceed 0.068.
4. **Training process**: In Supplementary Fig. S3, we present the molecular reconstruction rate and validity rate on the training set during model training. As the training epoch progresses, the molecular reconstruction rate on the training set steadily increases, reaching nearly 100% after approximately 50 epochs. Conversely, the validity rate of the model on the training set initially decreases, followed by an upward trend, eventually stabilizing at around 70%.

The “Best Model” was selected based on a high value of the sum of reconstruction and validity rates. In this case, the best model, exhibiting a training reconstruction rate of 97.95% and a training validity rate of 95.02%, was chosen for optimizing ligand molecules.

## 4 Conclusion and future work

This study explores the value of ACs in optimizing ligand bioactivity. We introduce a novel approach, termed OLB-AC, which utilizes DGL and ACs to optimize ligand bioactivities. OLB-AC integrates graph embedding networks, adversarial/generative learning, and an AGRN to simultaneously optimize classification/regression loss, adversarial/generative loss, and reconstruction/optimization loss. Through extensive benchmark testing, we demonstrated that OLB-AC can optimize ligand bioactivity, enhance the inhibitory efficacy of molecules, and reduce their toxicity. It also outperforms current state-of-the-art methods in terms of bioactivity prediction, which serves as the foundation for optimization. Visualizing the generated ligands with OLB-AC extends our understanding of adversarial samples and the modeling of ACs. Additionally, OLB-AC exhibits potential for guiding the optimization of ligand bioactivities and can be further extended to optimize ligand properties. Nevertheless, optimizing ligand bioactivity using ACs presents numerous challenges, and our work represents just the beginning of this exploration. In the future, we plan to explore more modeling techniques, such as large language models for ligands, and consider a wider range of AC types.

## Supplementary Material

btae365_Supplementary_Data

## Data Availability

The data underlying this article are available in https://github.com/Yueming-Yin/OLB-AC.
