# Supplementary material for: OLB-AC: toward optimizing ligand bioactivities through deep graph learning and activity cliffs
Source: Bioinformatics. 2024 Jun 18;40(6):btae365. doi: 10.1093/bioinformatics/btae365 (PMC11208724; doi:10.1093/bioinformatics/btae365)
Supplement: btae365_Supplementary_Data [file btae365_supplementary_data.pdf]

# **Supporting Information of**
# OLB-AC: Towards Optimizing Ligand Bioactivities Through Deep Graph Learning and Activity Cliffs

Yueming Yin[1,2], Haifeng Hu *[1], Jitao Yang[1], Chun Ye[1], Wilson Wen Bin Goh [4,5], Adams Wai-Kin Kong[2], and Jiansheng Wu †[6]

[1]School of Telecommunications and Information Engineering, Nanjing University of Posts and Telecommunications, Nanjing 210003, China.
[2]School of Computer Science and Engineering, Nanyang Technological University 639798, Singapore.
[4]Lee Kong Chian School of Medicine, Nanyang Technological University 637551, Singapore.
[5]Center for Biomedical Informatics, 636921, Singapore.
[6]School of Computer Science, Nanjing University of Posts and Telecommunications, Nanjing 210023, China.

June 7, 2024

*Corresponding author: huhf@njupt.edu.cn
†Corresponding author: jansen@njupt.edu.cn

# Contents


- Tables
  - ☐ Table S1: Notations . . . . . . . . . . . . . . . . . . . . . . . . . . . . . . . . . . . . . . . . . . . . . . . Page S-5
  - ☐ Table S2: Atomic and Bond Chemical Features, activation functions and loss functions in model learning . . . . . . . . . . . . . . . . . . . . . . . . . . . . . . . . . . . . . . . . . . . Page S-6
  - ☐ Table S3: Novel Transformations of OLB-AC Optimized Molecules . . . . Page S-7
  - ☐ Table S4: Optimizing Ligand bioactivities through OLB-AC . . . . . . . . . . . Page S-8
  - ☐ Table S5: Enhancing ligand inhibitory efficacy through OLB-AC . . . . . . . Page S-9
  - ☐ Table S6: Reducing ligand toxicity through OLB-AC . . . . . . . . . . . . . . . . Page S-10
  - ☐ Table S7: Reverse optimization on ligand bioactivities by OLB-AC . . . . Page S-11
  - ☐ Table S8: Comparison of the square of Pearson correlation coefficient $r^2$ index on the ligand bioactivity prediction benchmark (Activity-33) . . . . . . . . . . . . Page S-12
  - ☐ Table S9: Comparison of the root mean square error (RMSE) on the ligand bioactivity prediction benchmark (Activity-33) . . . . . . . . . . . . . . . . . . . . . . . . . . . Page S-13
  - ☐ Table S10: Comparison of classification performance indexes on ADMET-25 benchmark . . . . . . . . . . . . . . . . . . . . . . . . . . . . . . . . . . . . . . . . . . . . . . . . . . . . . . . . . . . Page S-14
  - ☐ Table S11: Comparison of model parameters, training time, and performance in Task 7 of Activity-33 . . . . . . . . . . . . . . . . . . . . . . . . . . . . . . . . . . . . . . . . . . . . Page S-15
- Figures
  - ☐ Figure S1: The predicted bioactivity distributions between optimized molecules and the original low-activity molecules in the Task 7 of Activity-33 . . . . Page S-16
  - ☐ Figure S2: Ablation study on hyperparameters in the Task 7 of Activity-33 . . Page S-16
  - ☐ Figure S3: The reconstruction rate and validity rate during training epochs in Task 12 of Activity-33. . . . . . . . . . . . . . . . . . . . . . . . . . . . . . . . . . . . . . . . . . . . . . . Page S-17
- Algorithms
  - ☐ Algorithm S1: Molecular embedding algorithm of Attentive FP . . . . . . . Page S-18
  - ☐ Algorithm S2: Molecular graph generation algorithm of AGRNs . . . . . . Page S-19
  - ☐ Algorithm S3: Optimizing ligand bioactivity through OLB-AC . . . . . . . Page S-20
- Texts
  - ☐ Text S1: Performance indexes . . . . . . . . . . . . . . . . . . . . . . . . . . . . . . . . . . . . Page S-21
  - ☐ Text S2: Enhancing inhibitory efficacy and reducing toxicity through OLB-AC's ligand bioactivity optimization . . . . . . . . . . . . . . . . . . . . . . . . . . . . . . . . . . . . Page S-23

Table S1: Notations

| | |
|---|---|
| ***Functions*** | |
| $\mathrm{CE}$ | Cross-entropy loss |
| $\mathrm{dropout}$ | Randomly drop part of neural network nodes in the training stage |
| $\mathrm{D}$ | Discrepancy function |
| $\mathrm{elu}$ | Exponential linear unit |
| $\mathrm{E}$ | Graph encoder |
| $\mathrm{G}$ | Graph decoder |
| $\mathrm{GRU}$ | Gated recurrent unit |
| $\mathrm{L}$ | Error function between predictions and assays |
| $\mathrm{leaky\_relu}$ | Leaky rectified linear unit |
| $\mathcal{L}_{AFSE}$ | Adversarial feature subspace enhancement loss function |
| $\mathcal{L}_{Bio.}$ | Biological property loss function |
| $\mathcal{L}_{Recon.}$ | Molecular reconstruction loss function |
| $\mathcal{L}_{Val.}$ | Chemical validity loss function |
| $\mathrm{N}$ | Multi-layer perceptron |
| $\phi_a$, $\phi_b$ | Mapping function of initial atomic/bond feature |
| $\mathrm{relu}$ | Rectified linear unit |
| $\mathrm{softmax}$ | Softmax activate function |
| $\mathrm{Valid}$ | Validity function |
| $\mathrm{WCE}$ | Weighted cross-entropy loss |
| ***Indices*** | |
| $i^*$ | Index of the key atom |
| $k_i$ | Index of the original symbol of atom $\mathbf{a}_i$ |
| $k^*$ | Index of the replaced chemical element |
| $L$ | Number of atomic message passing steps ($l \in \{1, \cdots, L\}$) |
| $N_a$ | Number of atoms in a molecule ($i \in \{1, \cdots, N_a\}$) |
| $N_s$ | Number of element types ($k \in \{1, \cdots, N_s\}$) |
| $T$ | Number of molecular massage passing steps ($t \in \{1, \cdots, T\}$) |
| ***Matrices*** | |
| $\mathbf{W}$ | Weight matrices of independent one-layer neural networks, their column and row dimensions depend on their respective input and output vectors |
| ***Operators*** | |
| $[\cdot]$ | Matrix indexing |
| $[\cdot, \cdot]$ | Row concatenation |
| $\|\| \cdot \|\|$ | $l_2$-norm |
| $\nabla$ | Gradient operator |
| ***Parameters*** | |

| | |
|---|---|
| $\lambda_1$ | Balance coefficient between the biological property loss and the AFSE loss |
| $\lambda_2$ | Balance coefficient between the representation learning and the ligand bioactivity optimization |

***Sets***

| | |
|---|---|
| $\mathcal{B}_i$, $\hat{\mathcal{B}}_i$, $\widetilde{\mathcal{B}}_i$ | Set of $\mathbf{b}_{i,j}$, $\hat{\mathbf{b}}_{i,j}$ and $\widetilde{\mathbf{b}}_{i,j}$ for all $j \in \{1, \cdots, N_{N(i)}\}$, respectively |
| $\mathcal{H}$ | Set of $\mathbf{h}_i$ for all $i \in \{1, \cdots, N_a\}$ |
| $\mathcal{K}_i$ | Set of original and confusing element on the $i$-th atom |
| $\mathcal{K}_i^*$ | Set of candidate element on the $i$-th atom |
| $N(i)$ | index set of adjacent atom $j \in N(i)$ of the $i$-th atom |

***Scalars***

| | |
|---|---|
| $\mathbf{a}_{i,k}$, $\hat{\mathbf{a}}_{i,k}$, $\widetilde{\mathbf{a}}_{i,k}$ | True/reconstructed/optimized probability of the $i$-th atom belonging to the $k$-th element |
| $\eta$ | Learning rate of N |
| $\gamma_i$ | Projection of atomic embedding on molecular embedding |
| $P_{\mathbf{f}}(s\|a)$ | The posterior probability that the atom $a$ is predicted to be the element $s$ According to the embedding $\mathbf{f}$, equal to $\hat{\mathbf{a}}_{i=a,k=s}$ |
| $P_{\mathbf{f}+\mathbf{d}}(s\|a)$ | The posterior probability that the atom $a$ is predicted to be the element $s$ according to the embedding $\mathbf{f}+\mathbf{d}$, equal to $\widetilde{\mathbf{a}}_{i=a,k=s}$ |
| $\varepsilon$ | Small positive rational number |
| $w_{ji}$ | Attention weight between the $i$-th atom and its adjacent atoms $j \in N(i)$ |
| $y$ | The experimentally determined ligand bioactivity or property values through chemical wet experiments |
| $\hat{y}$ | Predicted ligand bioactivities or properties |

***Column Vectors***

| | |
|---|---|
| $\mathbf{a}_i$, $\hat{\mathbf{a}}_i$, $\widetilde{\mathbf{a}}_i$ | True/reconstructed/optimized chemical feature vector of the $i$-th atom |
| $\mathbf{b}_{i,j}$, $\hat{\mathbf{b}}_{i,j}$, $\widetilde{\mathbf{b}}_{i,j}$ | True/reconstructed/optimized chemical feature vector of the bond between the $i$-th and the $j$-th atom |
| $\mathbf{c}$ | Bias vector of independent one-layer neural networks |
| $\mathbf{C}_i^l$ | Context feature of the $i$-th atom |
| $\mathbf{d}$ | Adversarial perturbation generated by AFSE algorithm |
| $\mathbf{f}$ | Molecular embedding |
| $\mathbf{f}_t$ | Molecular embedding at the $t$-th training step |
| $\mathbf{g}_i$ | Atomic embedding of the $i$-th atom for molecular reconstruction and optimization |
| $\mathbf{g}_i^t$, $\mathbf{g}_i^l$ | $\mathbf{g}_i$ at the $t$-th molecular or the $l$-th atomic message passing step |
| $\mathbf{g}_{ji}$ | Atomic embedding of the neighbor atom $j \in N(i)$ of the $i$-th atom for molecular reconstruction and optimization |
| $\mathbf{g}_{ji}^t$, $\mathbf{g}_{ji}^l$ | $\mathbf{g}_{ji}$ at the $t$-th molecular or the $l$-th atomic message passing step |
| $\mathbf{h}_i$ | Atomic embedding of the $i$-th atom for molecular bioactivity and property prediction |
| $\mathbf{h}_i^t$, $\mathbf{h}_i^l$ | $\mathbf{h}_i$ at the $t$-th molecular or the $l$-th atomic message passing step |

| | |
|---|---|
| $\mathbf{h}_{ji}$ | Atomic embedding of the neighbor atom $j \in N(i)$ of the $i$-th atom for molecular bioactivity and property prediction |
| $\mathbf{h}^t_{ji}$, $\mathbf{h}^l_{ji}$ | $\mathbf{h}_{ji}$ at the $t$-th molecular or the $l$-th atomic message passing step |
| $\mathbf{r}$ | Random vector |
| $\mathbf{r}_i$ | The relationship embedding between the $i$-th atom and the whole molecule |

Table S2: Atomic and Bond Chemical Features, activation functions ($\phi_a$, $\phi_b$) and loss functions ($L_a$, $L_b$) in model learning.

| atom feature | type | size | description | $\phi_a$ | $L_a$ |
|---|---|---|---|---|---|
| atomic symbol | one-hot | 16 | [B, C, N, O, F, Si, P, S, Cl, As, Se, Br, Te, I, At, metal] | Softmax | WCE [a] |
| degree | one-hot | 6 | number of covalent bonds [0,1,2,3,4,5] | Softmax | CE [b] |
| formal charge | integer | 1 | electrical charge | / | MSE [c] |
| radical electrons | integer | 1 | number of radical electrons | ReLU | MSE |
| hybridization | one-hot | 6 | [sp, $sp^2$, $sp^3$, $sp^3d$, $sp^3d^2$, other] | Softmax | CE |
| aromaticity | binary | 1 | whether the atom is part of an aromatic system [0/1] | Sigmoid | CE |
| hydrogens | one-hot | 5 | number of connected hydrogens [0,1,2,3,4] | Softmax | CE |
| chirality | binary | 1 | whether the atom is chiral center [0/1] | Sigmoid | CE |
| chirality type | binary | 2 | [R,S] | Sigmoid | CE |
| **bond feature** | **type** | **size** | **description** | $\phi_b$ | $L_b$ |
| bond type | one-hot | 4 | [single, double, triple, aromatic] | Softmax | CE |
| conjugation | binary | 1 | whether the bond is conjugated [0/1] | Sigmoid | CE |
| ring | binary | 1 | whether the bond is in ring [0/1] | Sigmoid | CE |
| stereo | one-hot | 4 | [StereoNone, StereoAny, StereoZ, StereoE] | Softmax | CE |

[a] "WCE" means the weighted cross-entropy loss. [b] "CE" means cross-entropy loss. [c] "MSE" means mean square error.

Table S3: The drug targets, common parts, novel transformations (not found in their training sets), and properties between original and OLB-AC optimized molecules.

| Targets | Common Parts | Original Properties | Novel Transformations | Optimized Properties |
|---|---|---|---|---|
| Human sphingosine 1-phosphate receptor (P21453) | 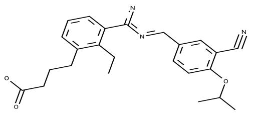 | Activity [a]: 8.6002<br>QED [b]: 0.4766<br>SA [c]: 2.7684<br>logP [d]: 5.5007 | 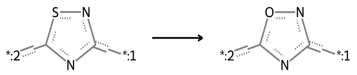 | Activity: 9.6995 (++)<br>QED: 0.5177 (+)<br>SA: 2.5739 (+)<br>logP: 5.0322 (+) |
| Human orexin type 2 receptor (O43614) | 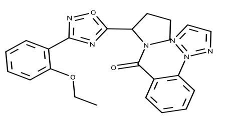 | Activity: 6.9788<br>QED: 0.4419<br>SA: 3.0856<br>logP: 3.8324 | 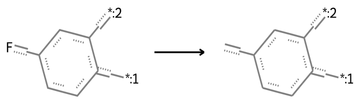 | Activity: 8.3979 (++)<br>QED: 0.4431 (+)<br>SA: 3.0689 (+)<br>logP: 4.0017 (−) |
| | 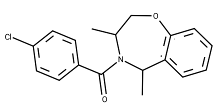 | Activity: 5.5229<br>QED: 0.7706<br>SA: 3.0386<br>logP: 4.4634 | 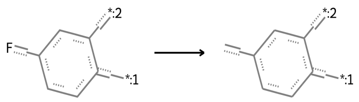 | Activity: 6.8239 (++)<br>QED: 0.7629 (−)<br>SA: 3.0154 (−)<br>logP: 4.6327 (−) |
| | 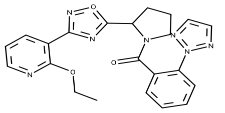 | Activity: 7.1249<br>QED: 0.4410<br>SA: 3.2419<br>logP: 3.2274 | 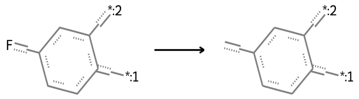 | Activity: 8.1549 (++)<br>QED: 0.4439 (+)<br>SA: 3.2251 (+)<br>logP: 3.3967 (+) |
| | 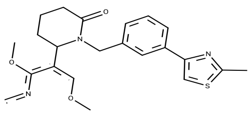 | Activity: 6.2083<br>QED: 0.5908<br>SA: 3.1402<br>logP: 4.1795 | 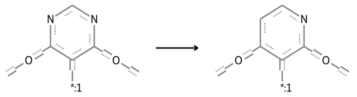 | Activity: 7.8861 (++)<br>QED: 0.5706 (−)<br>SA: 3.0246 (+)<br>logP: 4.7845 (−) |

[a] Activity: ligands' bioactivity to targets, measured by pIC50, pEC50, pKi, or pKd. [b] QED: quantitative estimation of drug-likeness. QED ranges from 0 to 1, with higher values indicating higher drug-like properties. [c] SA: synthesizability. SA ranges from 1 to 10, with closer values to 1 indicating easier synthesis. [d] LogP: oil-water partition coefficient. Drugs are well absorbed when LogP values are between 0 and 3.

## Table S4: Optimizing ligand bioactivities through OLB-AC.

| Targets | Anchor Ligands | Anchor Properties | Optimized Ligands | Optimized Properties |
|---|---|---|---|---|
| Human sphingosine 1-phosphate receptor (P21453) | 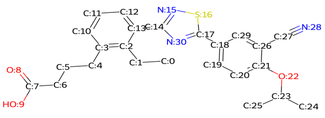 | Activity [a]: 8.6002<br>QED [b]: 0.4766<br>SA [c]: 2.7684<br>logP [d]: 5.5007 | 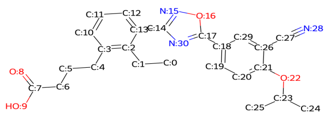 | Atom#16: S→O<br>Activity: 9.6995 (++)<br>QED: 0.5177 (+)<br>SA: 2.5739 (+)<br>logP: 5.0322 (+) |
| | 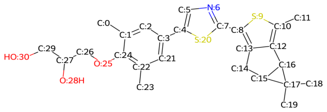 | Activity: 7.4225<br>QED: 0.5158<br>SA: 4.1048<br>logP: 5.4916 | 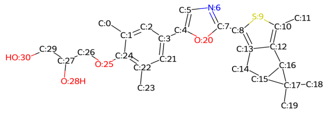 | Atom#20: S→O<br>Activity: 9.0000 (++)<br>QED: 0.5638 (+)<br>SA: 4.1124 (−)<br>logP: 5.0231 (+) |
| | 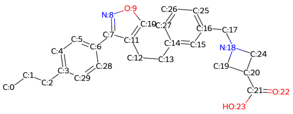 | Activity: 6.6576<br>QED: 0.6539<br>SA: 2.4926<br>logP: 4.5761 | 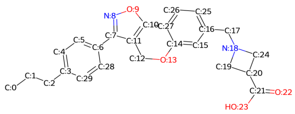 | Atom#13: C→O<br>Activity: 8.8861 (++)<br>QED: 0.6575 (+)<br>SA: 2.5408 (−)<br>logP: 4.3699 (+) |
| Human orexin type 2 receptor (O43614) | 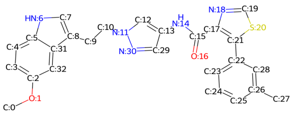 | Activity: 7.5229<br>QED: 0.3473<br>SA: 2.6436<br>logP: 5.2999 | 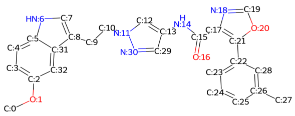 | Atom#20: S→O<br>Activity: 8.6990 (++)<br>QED: 0.3769 (+)<br>SA: 2.6295 (−)<br>logP: 4.8314 (+) |
| | 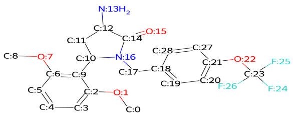 | Activity: 5.6882<br>QED: 0.7903<br>SA: 3.2084<br>logP: 3.4033 | 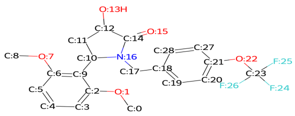 | Atom#13: N→O<br>Activity: 7.2291 (++)<br>QED: 0.7889 (−)<br>SA: 3.1571 (+)<br>logP: 3.4369 (−) |
| | 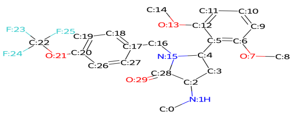 | Activity: 5.2358<br>QED: 0.7352<br>SA: 3.2157<br>logP: 3.6640 | 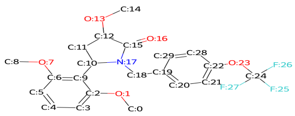 | Atom#1: N→O<br>Activity: 6.7747 (++)<br>QED: 0.6690 (−)<br>SA: 3.2724 (−)<br>logP: 4.0910 (−) |
| Human dopamine receptor (P14416) | 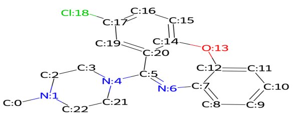 | Activity: 7.6778<br>QED: 0.7370<br>SA: 2.2952<br>logP: 3.7714 | 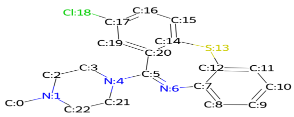 | Atom#13: O→S<br>Activity: 9.3098 (++)<br>QED: 0.7164 (−)<br>SA: 2.4025 (−)<br>logP: 4.1303 (−) |
| | 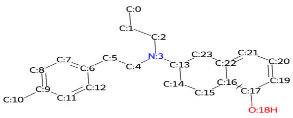 | Activity: 6.7696<br>QED: 0.8451<br>SA: 2.6875<br>logP: 4.5126 | 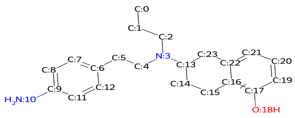 | Atom#10: C→N<br>Activity: 8.1675 (++)<br>QED: 0.7950 (−)<br>SA: 2.7957 (−)<br>logP: 3.7864 (+) |
| | 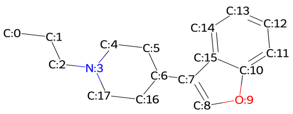 | Activity: 5.7545<br>QED: 0.8091<br>SA: 2.1075<br>logP: 4.0222 | 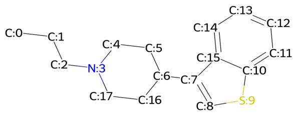 | Atom#9: O→S<br>Activity: 7.1337 (++)<br>QED: 0.7847 (−)<br>SA: 2.0823 (+)<br>logP: 4.4907 (−) |

[a] Activity: ligands' bioactivity to targets, measured by p-values of IC50, EC50, Ki, or Kd. [b] QED: quantitative estimation of drug-likeness. QED ranges from 0 to 1, with higher values indicating higher drug-like properties. [c] SA: synthesizability. SA ranges from 1 to 10, with closer values to 1 indicating easier synthesis. [d] LogP: oil-water partition coefficient. Drugs are well absorbed when LogP values are between 0 and 3.

# Table S5: Enhancing ligand inhibitory efficacy through OLB-AC.

| Targets | Anchor Ligands | Anchor Properties | Optimized Ligands | Optimized Properties |
|---|---|---|---|---|
| CYP1A2 Inhibitor | 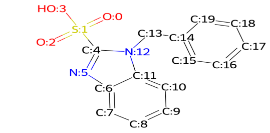 | Is Inhibitor [a]: No<br>QED [b]: 0.7507<br>SA [c]: 1.9675<br>logP [d]: 2.3313 | 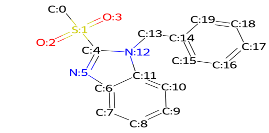 | Atom#3: O→C<br>Is Inhibitor: Yes<br>QED: 0.7430 (−)<br>SA: 1.8747 (+)<br>logP: 2.4881 (=) |
| | 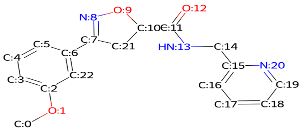 | Is Inhibitor: No<br>QED: 0.9151<br>SA: 2.6567<br>logP: 1.8996 | 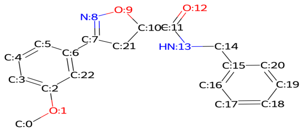 | Atom#20: N→C<br>Is Inhibitor: Yes<br>QED: 0.9228 (+)<br>SA: 2.4424 (+)<br>logP: 2.5046 (=) |
| CYP2C9 Inhibitor | 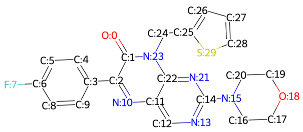 | Is Inhibitor: No<br>QED: 0.5026<br>SA: 2.5278<br>logP: 2.9390 | 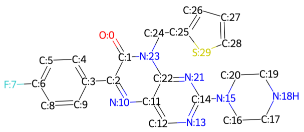 | Atom#18: O→N<br>Is Inhibitor: Yes<br>QED: 0.5447 (+)<br>SA: 2.6015 (−)<br>logP: 2.5120 (=) |
| | 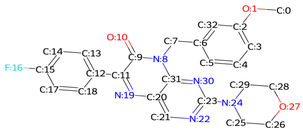 | Is Inhibitor: No<br>QED: 0.4653<br>SA: 2.4246<br>logP: 2.8861 | 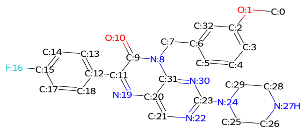 | Atom#27: O→N<br>Is Inhibitor: Yes<br>QED: 0.5041 (+)<br>SA: 2.4922 (−)<br>logP: 2.4591 (=) |
| CYP3A4 Inhibitor | 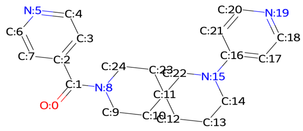 | Is Inhibitor: No<br>QED: 0.8458<br>SA: 2.8367<br>logP: 2.9994 | 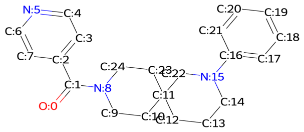 | Atom#19: N→C<br>Is Inhibitor: Yes<br>QED: 0.8412 (−)<br>SA: 2.6357 (+)<br>logP: 3.6044 (−) |
| | 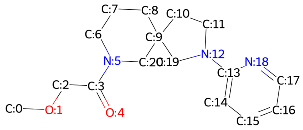 | Is Inhibitor: No<br>QED: 0.8467<br>SA: 3.2989<br>logP: 1.5469 | 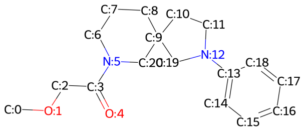 | Atom#18: N→C<br>Is Inhibitor: Yes<br>QED: 0.8542 (+)<br>SA: 3.1168 (+)<br>logP: 2.1519 (=) |

[a] Is Inhibitor: whether the ligands can inhibit the functions of targets, with “Yes” indicating can inhibit, while “No” can not. [b] QED: quantitative estimation of drug-likeness. QED ranges from 0 to 1, with higher values indicating higher drug-like properties. [c] SA: synthesizability. SA ranges from 1 to 10, with closer values to 1 indicating easier synthesis. [d] LogP: oil-water partition coefficient. Drugs are well absorbed when LogP values are between 0 and 3.

Table S6: Reducing ligand toxicity through OLB-AC.

| Targets | Anchor Ligands | Anchor Properties | Optimized Ligands | Optimized Properties |
|---|---|---|---|---|
| | 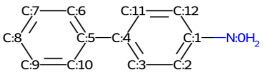 | Toxicity [a]: High<br>QED [b]: 0.6155<br>SA [c]: 1.2681<br>logP [d]: 3.8570 | 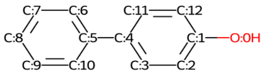 | Atom#0: N→O<br>Toxicity: Non-Toxic<br>QED: 0.6969 (+)<br>SA: 1.2254<br>logP: 3.0592 (+) |
| NR-AhR Toxicity | 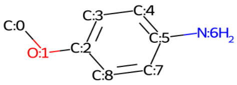 | Toxicity: High<br>QED: 0.5630<br>SA: 1.4461<br>logP: 2.4220 | 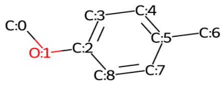 | Atom#6: N→C<br>Toxicity: Non-Toxic<br>QED: 0.5532<br>SA: 1.0100 (+)<br>logP: 2.0036 |
| | 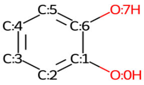 | Toxicity: High<br>QED: 0.3762<br>SA: 1.8671<br>logP: 1.4854 | 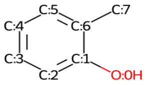 | Atom#7: O→C<br>Toxicity: Non-Toxic<br>QED: 0.5359 (++)<br>SA: 1.4050 (+)<br>logP: 1.7006 |
| | 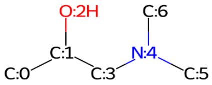 | Toxicity: High<br>QED: 0.5285<br>SA: 2.7983<br>logP: -0.0712 | 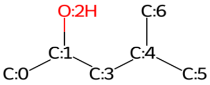 | Atom#4: N→C<br>Toxicity: Non-Toxic<br>QED: 0.5586 (+)<br>SA: 2.6752<br>logP: 1.4133 (++) |
| NR-ER Toxicity | 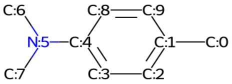 | Toxicity: High<br>QED: 0.5694<br>SA: 1.3958<br>logP: 2.0610 | 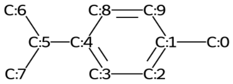 | Atom#5: N→C<br>Toxicity: Non-Toxic<br>QED: 0.5533<br>SA: 1.2512 (+)<br>logP: 3.1184 (−) |
| | 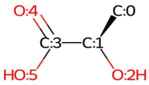 | Toxicity: High<br>QED: 0.4539<br>SA: 2.3257<br>logP: -0.5482 | 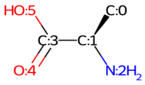 | Atom#2: O→N<br>Toxicity: Non-Toxic<br>QED: 0.4514<br>SA: 2.3196<br>logP: -0.5818 |
| | 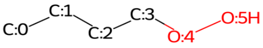 | Toxicity: High<br>QED: 0.3211<br>SA: 2.3758<br>logP: 1.2761 | 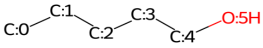 | Atom#4: N→C<br>Toxicity: Non-Toxic<br>QED: 0.5133 (++)<br>SA: 1.5665 (+)<br>logP: 1.1689 |
| AMES Toxicity | 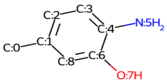 | Toxicity: High<br>QED: 0.4030<br>SA: 1.8514<br>logP: 1.2828 | 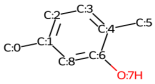 | Atom#5: N→C<br>Toxicity: Non-Toxic<br>QED: 0.5577 (++)<br>SA: 1.5860 (+)<br>logP: 2.0090 |
| | 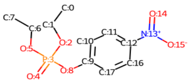 | Toxicity: High<br>QED: 0.4312<br>SA: 2.3371<br>logP: 3.1547 | 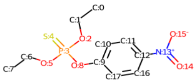 | Atom#4: O→S<br>Toxicity: Non-Toxic<br>QED: 0.4360<br>SA: 2.5031<br>logP: 3.2711 |

[a] Toxicity: ligands' ability to activate the toxic functions of targets. [b] QED: quantitative estimation of drug-likeness. QED ranges from 0 to 1, with higher values indicating higher drug-like properties. [c] SA: synthesizability. SA ranges from 1 to 10, with closer values to 1 indicating easier synthesis. [d] LogP: oil-water partition coefficient. Drugs are well absorbed when LogP values are between 0 and 3.

Table S7: Reverse optimization on ligand bioactivities by OLB-AC.

| Targets | Anchor Ligands | Anchor Properties | Optimized Ligands | Optimized Properties |
|---|---|---|---|---|
| Human sphingosine 1-phosphate receptor (P21453) | 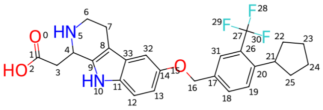 | Activity [a]: 10.2218<br>QED [b]: 0.4062<br>SA [c]: 3.2172<br>logP [d]: 6.0848 | 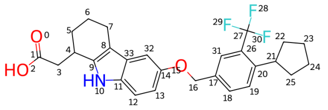 | Atom#5: N→C<br>Activity: 8.7696 (−−)<br>QED: 0.3960 (−)<br>SA: 3.1167 (+)<br>logP: 7.3179 (−) |
| | 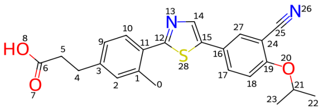 | Activity: 9.7447<br>QED: 0.5630<br>SA: 2.5247<br>logP: 5.4616 | 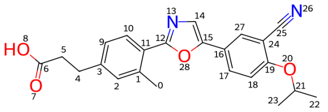 | Atom#28: S→O<br>Activity: 8.2924 (−−)<br>QED: 0.6102 (+)<br>SA: 2.4828 (+)<br>logP: 4.9931 (+) |
| Human orexin type 2 receptor (O43614) | 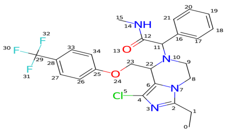 | Activity: 8.0458<br>QED: 0.4875<br>SA: 3.5422<br>logP: 5.0407 | 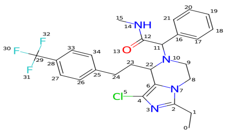 | Atom#24: O→C<br>Activity: 6.9706 (−−)<br>QED: 0.4558 (−)<br>SA: 3.5376<br>logP: 5.5945 (−) |
| | 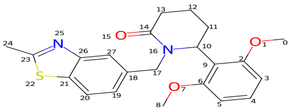 | Activity: 7.9031<br>QED: 0.6170<br>SA: 2.8575<br>logP: 4.8757 | 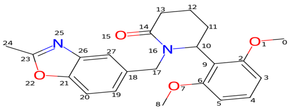 | Atom#22: S→O<br>Activity: 6.6517 (−−)<br>QED: 0.6561 (+)<br>SA: 2.8836<br>logP: 4.4072 (+) |
| Human dopamine receptor (P14416) | 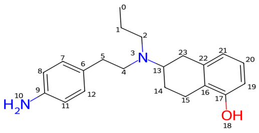 | Activity: 8.1675<br>QED: 0.7950<br>SA: 2.7957<br>logP: 3.7864 | 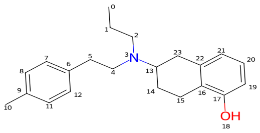 | Atom#10: N→C<br>Activity: 6.7696 (−−)<br>QED: 0.8451 (+)<br>SA: 2.6875 (+)<br>logP: 4.5126 (−) |
| | 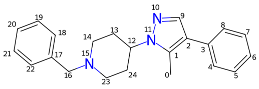 | Activity: 6.8539<br>QED: 0.6910<br>SA: 2.0137<br>logP: 4.6956 | 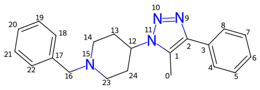 | Atom#9: C→N<br>Activity: 5.7959 (−−)<br>QED: 0.7214 (+)<br>SA: 2.0890<br>logP: 4.0906 (+) |

[a] Activity: ligands' bioactivity to targets, measured by p-values of IC50, EC50, Ki, or Kd. [b] QED: quantitative estimation of drug-likeness. QED ranges from 0 to 1, with higher values indicating higher drug-like properties. [c] SA: synthesizability. SA ranges from 1 to 10, with closer values to 1 indicating easier synthesis. [d] LogP: oil-water partition coefficient. Drugs are well absorbed when LogP values are between 0 and 3.

Table S8: Comparison of the square of Pearson correlation coefficient $r^2$ index on the ligand bioactivity prediction benchmark (Activity-33). Baseline results are taken from [1].

| Methods | | Fingerprint-based | | Graph-based | | | | | | | | |
|---|---|---|---|---|---|---|---|---|---|---|---|---|
| Dataset sizes | Task IDs [a] | RF | MLP | GATs | GINs | Neural FP | Weave | MPNN | Attentive FP | Uni-Mol | AFSE [b] | OLB-AC |
| Small (200 ∼ 400) | 1 | 0.0031 | -0.0812 | 0.1576 | 0.1347 | 0.0384 | 0.2785 | 0.1551 | 0.2508 | 0.2983 | 0.4347 | **0.4621** |
| | 2 | -0.0288 | -0.1527 | 0.0348 | 0.0015 | 0.0558 | 0.0564 | 0.0246 | 0.1235 | **0.3117** | 0.1030 | 0.2701 |
| | 3 | 0.3735 | 0.3073 | 0.1764 | 0.2235 | 0.1260 | 0.3217 | 0.1885 | 0.1817 | 0.3637 | 0.2774 | **0.4399** |
| | 4 | 0.4473 | 0.1627 | 0.1207 | 0.0645 | 0.0032 | 0.1132 | 0.0079 | 0.2786 | 0.3995 | 0.4660 | **0.4695** |
| | 5 | 0.4025 | 0.4868 | 0.4240 | 0.2425 | 0.3089 | 0.5272 | 0.4724 | 0.5397 | 0.4793 | 0.6423 | **0.6793** |
| | 6 | 0.1486 | 0.2175 | 0.3849 | 0.3216 | 0.0490 | 0.3383 | 0.3347 | 0.2534 | 0.3752 | 0.2022 | **0.4490** |
| | 7 | 0.2071 | 0.1775 | 0.2871 | 0.0360 | 0.0437 | 0.2871 | 0.0008 | 0.1866 | 0.2770 | 0.5079 | **0.5842** |
| Medium (1000 ∼ 2000) | 8 | 0.4832 | 0.5206 | 0.4844 | 0.0336 | 0.4764 | 0.4455 | 0.4215 | 0.6272 | 0.6138 | 0.6201 | **0.6306** |
| | 9 | 0.2412 | 0.0807 | 0.3089 | 0.1791 | 0.1306 | 0.4217 | 0.3232 | 0.5133 | 0.2988 | 0.5018 | **0.5292** |
| | 10 | 0.4958 | 0.4981 | 0.4086 | 0.3585 | 0.0223 | 0.5076 | 0.5086 | 0.4764 | 0.4356 | 0.5102 | **0.5547** |
| | 11 | 0.1486 | 0.2774 | 0.0896 | 0.0022 | 0.0200 | 0.2701 | 0.0820 | 0.3614 | 0.2344 | 0.3669 | **0.3979** |
| | 12 | 0.4158 | 0.5426 | 0.4568 | 0.4352 | 0.2020 | 0.5118 | 0.3565 | 0.5841 | 0.5656 | 0.5926 | **0.5951** |
| | 13 | 0.2183 | 0.2098 | 0.2215 | 0.1274 | 0.0008 | 0.2760 | 0.3014 | 0.3690 | 0.3297 | 0.3702 | **0.4020** |
| | 14 | 0.4562 | 0.6055 | 0.3434 | 0.2880 | 0.3178 | 0.5818 | 0.6033 | 0.5286 | 0.5435 | 0.6144 | **0.6254** |
| | 15 | 0.0473 | 0.2227 | 0.3096 | 0.0444 | 0.0066 | 0.3138 | 0.2898 | 0.2684 | 0.2014 | 0.3943 | **0.4039** |
| | 16 | 0.4797 | 0.5371 | **0.6994** | 0.3929 | 0.1976 | 0.5995 | 0.4494 | 0.6422 | 0.5837 | 0.6416 | 0.6315 |
| | 17 | 0.1485 | 0.1284 | 0.1870 | 0.0364 | 0.1044 | 0.2325 | 0.2571 | 0.2400 | 0.2079 | 0.3032 | **0.3090** |
| Large (2000 ∼ 4000) | 18 | 0.5227 | 0.4544 | 0.5506 | 0.4098 | 0.4052 | 0.5500 | 0.5098 | 0.5768 | **0.5958** | 0.5766 | 0.5682 |
| | 19 | 0.3862 | 0.5097 | 0.2379 | 0.4676 | 0.0390 | 0.5252 | 0.5455 | 0.5440 | 0.4443 | 0.5188 | **0.5585** |
| | 20 | 0.3896 | 0.4708 | 0.5296 | 0.2265 | 0.0781 | 0.4225 | 0.4700 | 0.5589 | 0.4192 | 0.5152 | **0.5664** |
| | 21 | 0.3458 | 0.4238 | 0.5636 | 0.4297 | 0.0779 | 0.4964 | 0.4459 | 0.5164 | 0.4333 | 0.5173 | **0.5742** |
| | 22 | 0.3989 | 0.4682 | 0.0156 | 0.5024 | 0.0069 | 0.4119 | 0.5397 | 0.5338 | 0.4527 | 0.5291 | **0.5915** |
| | 23 | 0.3698 | 0.4503 | 0.5138 | 0.3694 | 0.2724 | 0.4542 | 0.4429 | 0.5092 | 0.5160 | 0.5674 | **0.5851** |
| | 24 | 0.5592 | 0.6501 | **0.6855** | 0.4723 | 0.5400 | 0.5397 | 0.5773 | 0.6009 | 0.6046 | 0.6161 | 0.6297 |
| | 25 | 0.3530 | 0.4943 | 0.4338 | 0.3764 | 0.0947 | 0.4841 | 0.5319 | 0.5989 | 0.5640 | **0.7110** | 0.6178 |
| | 26 | 0.3762 | 0.4840 | 0.2426 | 0.3607 | 0.0844 | 0.4808 | 0.4895 | 0.5427 | 0.3796 | 0.5809 | **0.5941** |
| | 27 | 0.5059 | 0.5116 | 0.5117 | 0.4142 | 0.1699 | 0.5501 | 0.5210 | 0.4363 | 0.5311 | 0.5522 | **0.5573** |
| | 28 | 0.2662 | 0.2973 | 0.3673 | 0.1815 | 0.0095 | 0.3270 | 0.4229 | 0.3782 | 0.2870 | 0.4087 | **0.4714** |
| | 29 | 0.3362 | 0.3475 | 0.3723 | 0.3751 | 0.2645 | 0.5212 | 0.4113 | 0.5334 | 0.5042 | **0.6177** | 0.5710 |
| | 30 | 0.3411 | 0.4566 | 0.3516 | 0.2603 | 0.2193 | 0.4394 | 0.4495 | 0.3759 | 0.4214 | 0.3941 | **0.4687** |
| | 31 | 0.1818 | 0.2378 | 0.2528 | 0.0975 | 0.0056 | 0.3273 | 0.2788 | 0.4325 | 0.3720 | **0.4972** | 0.4454 |
| | 32 | 0.4178 | 0.5599 | 0.5018 | 0.2711 | 0.4874 | 0.5740 | 0.4962 | 0.5942 | 0.6088 | 0.5883 | **0.6148** |
| | 33 | 0.3402 | 0.5144 | 0.1259 | 0.1947 | 0.0214 | 0.5589 | 0.5171 | 0.5088 | 0.5090 | 0.5025 | **0.5614** |
| Average | | 0.3266 | 0.3658 | 0.3440 | 0.2525 | 0.1479 | 0.4165 | 0.3765 | 0.4444 | 0.4292 | 0.4922 | **0.5275** |

[a] Under the same dataset size, the larger the value of Task ID, the greater the difference in bioactivity distribution between test and training data. [b] Here, AFSE takes the Attentive FP as the backbone network.

Table S9: Comparison of the root mean square error (RMSE) on the ligand bioactivity prediction benchmark (Activity-33). Baseline results are taken from [1].

| Methods | | Fingerprint-based | | Graph-based | | | | | | | | |
|---|---|---|---|---|---|---|---|---|---|---|---|---|
| Dataset sizes | Task IDs [a] | RF | MLP | GATs | GINs | Neural FP | Weave | MPNN | Attentive FP | Uni-Mol | AFSE [b] | OLB-AC |
| Small (200 ∼ 400) | 1 | 0.7923 | 0.8251 | 3.2974 | 0.9876 | 0.9313 | 3.1691 | 2.1669 | 0.7396 | 0.7343 | 0.6619 | **0.6058** |
| | 2 | 0.6588 | 0.6974 | 2.3726 | 2.8024 | 0.8453 | 0.6281 | 0.6423 | 0.6755 | 0.9536 | 0.6618 | **0.5512** |
| | 3 | 0.8744 | 0.9194 | 2.5808 | 1.8494 | 1.0364 | 0.9577 | 1.0550 | 1.0046 | 1.6957 | 1.0085 | **0.8347** |
| | 4 | 0.6934 | 0.8534 | 2.1593 | 1.6084 | 0.9968 | 1.5625 | 0.9549 | 0.8392 | 0.7854 | 0.7126 | **0.6854** |
| | 5 | 0.9743 | 0.9030 | 1.5101 | 1.3409 | 1.0489 | 0.9088 | 0.9081 | 0.8997 | 1.0795 | 0.7391 | **0.6981** |
| | 6 | 0.9067 | 0.8692 | 5.8169 | 6.4834 | 1.1815 | 1.5556 | 0.8004 | 1.0454 | 1.6406 | 0.8763 | **0.7898** |
| | 7 | 0.8081 | 0.8231 | 2.9244 | 1.3547 | 0.8868 | 0.8604 | 2.4965 | 0.9050 | 2.1393 | 0.8799 | **0.7754** |
| Medium (1000 ∼ 2000) | 8 | 1.0396 | 1.0012 | 1.0352 | 1.7376 | 1.3659 | 1.1049 | 1.0965 | 0.9107 | 1.2747 | 0.8900 | **0.8892** |
| | 9 | 0.7157 | 0.7878 | 0.6948 | 0.7468 | 0.9933 | 0.6466 | 0.6751 | 0.5831 | 1.0641 | 0.5764 | **0.5623** |
| | 10 | 0.7205 | 0.7189 | 0.8416 | 0.8133 | 1.1050 | 0.7131 | 0.7778 | 0.7607 | 1.1501 | 0.7379 | **0.6843** |
| | 11 | 1.0742 | 0.9895 | 1.3577 | 1.1592 | 2.2731 | 1.2515 | 1.1135 | 1.0015 | 1.4632 | 0.9792 | **0.8959** |
| | 12 | 1.0993 | 0.9726 | 1.0587 | 1.2824 | 3.3874 | 1.0253 | 1.1728 | 0.9503 | 2.1155 | 0.9182 | **0.8802** |
| | 13 | 0.9217 | 0.9267 | 1.0367 | 0.9702 | 1.2523 | 0.9082 | 0.8727 | 0.8498 | 1.5328 | **0.8425** | 0.8628 |
| | 14 | 1.0521 | **0.8962** | 1.3644 | 1.2050 | 2.2825 | 0.9223 | 0.9147 | 0.9983 | 1.5634 | 0.9802 | 0.9452 |
| | 15 | 1.0773 | 0.9731 | 0.9103 | 1.0715 | 1.1052 | 0.9611 | 0.9809 | 0.9502 | 1.4546 | 0.8969 | **0.8774** |
| | 16 | 0.8529 | 0.8045 | **0.6797** | 0.9918 | 1.1245 | 0.7565 | 0.8829 | 0.7340 | 1.4395 | 0.7186 | 0.7412 |
| | 17 | 0.9673 | 0.9786 | 1.3270 | 1.0248 | 1.1565 | 0.9486 | 0.9430 | 0.9910 | 2.0926 | 0.9487 | **0.8705** |
| Large (2000 ∼ 4000) | 18 | 0.8457 | 0.9041 | 0.8534 | 0.9578 | 1.2707 | 0.8363 | 0.8822 | 0.8308 | 0.9866 | **0.8008** | 0.8315 |
| | 19 | 0.7147 | 0.6387 | 0.9206 | 0.6821 | 0.9096 | 0.7025 | 0.6279 | **0.6245** | 1.1508 | 0.6612 | **0.6245** |
| | 20 | 0.7663 | 0.7135 | 0.6908 | 0.8836 | 1.1462 | 0.7588 | 0.7580 | 0.7274 | 1.2363 | 0.6918 | **0.6546** |
| | 21 | 0.8177 | 0.7674 | 0.7087 | 0.7862 | 1.5485 | 0.7300 | 0.7843 | 0.7299 | 1.0362 | 0.7244 | **0.6699** |
| | 22 | 0.7686 | 0.7230 | 2.0991 | 0.7168 | 1.0593 | 0.7646 | 0.6942 | 0.7348 | 1.3339 | 0.7201 | **0.6726** |
| | 23 | 0.8965 | 0.8373 | 0.7906 | 0.8946 | 1.1637 | 0.8372 | 0.8751 | 0.8525 | 1.1831 | 0.7491 | **0.7431** |
| | 24 | 0.8200 | **0.7305** | 0.7522 | 0.9087 | 1.0100 | 0.9054 | 0.8243 | 0.7830 | 1.3751 | 0.7799 | 0.7793 |
| | 25 | 0.8922 | 0.7888 | 0.8556 | 0.9065 | 2.0447 | 0.9147 | 0.7715 | 0.7176 | 1.1796 | **0.5991** | 0.7008 |
| | 26 | 0.8378 | 0.7619 | 0.9261 | 0.9767 | 1.6084 | 0.7626 | 0.7914 | 0.7812 | 1.5150 | 0.7079 | **0.6930** |
| | 27 | 0.7396 | 0.7353 | 0.7363 | 0.8284 | 2.3014 | 0.7039 | 0.7623 | 0.8024 | 1.3805 | 0.8023 | **0.7006** |
| | 28 | 0.7668 | 0.7504 | 0.7126 | 1.0297 | 1.1610 | 0.7607 | 0.6978 | 0.7298 | 1.1148 | 0.7009 | **0.6811** |
| | 29 | 0.9279 | 0.9200 | 0.9637 | 0.9027 | 1.3375 | 0.7971 | 0.9070 | 0.7808 | 1.1350 | **0.7059** | 0.7583 |
| | 30 | 0.9414 | 0.8549 | 0.9454 | 1.0338 | 1.5637 | 0.9687 | 0.8782 | 0.9396 | 1.6588 | 0.9048 | **0.8501** |
| | 31 | 0.8930 | 0.8619 | 0.8529 | 0.9695 | 1.0982 | 0.9179 | 0.9075 | 0.7422 | 1.3911 | **0.7193** | 0.7519 |
| | 32 | 1.0531 | 0.9156 | 0.9711 | 1.2062 | 1.1339 | 0.9561 | 1.0094 | 0.9080 | 1.5548 | 0.8863 | **0.8616** |
| | 33 | 0.8012 | 0.6874 | 0.9424 | 0.9248 | 1.4976 | 0.6658 | 0.6848 | 0.7108 | 1.3430 | 0.6984 | **0.6594** |
| | Average | 0.8700 | 0.8343 | 1.3845 | 1.2739 | 1.3584 | 0.9655 | 0.9488 | 0.8253 | 1.3562 | 0.7843 | **0.7510** |

[a] Under the same dataset size, the larger the value of Task ID, the greater the difference in bioactivity distribution between test and training data. [b] Here, AFSE takes the Attentive FP as the backbone network.

Table S10: Comparison of classification performance indexes on ADMET-25 benchmark. Baseline results are taken from [2].

| Category | Model | AUC | | ACC | | MCC | | Specificity | | Sensitivity | |
|---|---|---|---|---|---|---|---|---|---|---|---|
| | | ADMETlab2.0 | OLB-AC | ADMETlab2.0 | OLB-AC | ADMETlab2.0 | OLB-AC | ADMETlab2.0 | OLB-AC | ADMETlab2.0 | OLB-AC |
| Absorption | Pgp-inhibitor | 0.922 | **0.923** | **0.867** | 0.857 | **0.723** | 0.714 | 0.844 | **0.906** | **0.882** | 0.801 |
| | Pgp-substrate | 0.840 | **0.923** | 0.768 | **0.857** | 0.538 | **0.714** | 0.705 | **0.906** | **0.828** | 0.801 |
| Distribution | BBB Penetration | **0.908** | 0.900 | **0.862** | 0.827 | **0.718** | 0.634 | **0.824** | 0.822 | **0.891** | 0.836 |
| Metabolism | CYP1A2 inhibitor | **0.928** | 0.923 | 0.852 | **0.857** | 0.704 | **0.714** | 0.848 | **0.906** | **0.857** | 0.801 |
| | CYP1A2 substrate | 0.737 | **0.900** | 0.649 | **0.827** | 0.298 | **0.634** | 0.632 | **0.822** | 0.667 | **0.836** |
| | CYP2C9 inhibitor | **0.919** | 0.900 | **0.841** | 0.827 | **0.671** | 0.634 | **0.823** | 0.822 | **0.878** | 0.836 |
| | CYP2C9 substrate | 0.725 | **0.904** | 0.707 | **0.798** | 0.386 | **0.619** | **0.776** | 0.723 | 0.606 | **0.904** |
| | CYP3A4 inhibitor | **0.921** | 0.904 | **0.832** | 0.798 | **0.659** | 0.619 | **0.825** | 0.723 | 0.841 | **0.904** |
| | CYP3A4 substrate | 0.776 | **0.912** | 0.713 | **0.823** | 0.437 | **0.647** | **0.820** | 0.810 | 0.608 | **0.836** |
| Toxicity | AMES Toxicity | 0.902 | **0.912** | 0.807 | **0.823** | 0.606 | **0.647** | 0.732 | **0.810** | **0.865** | 0.836 |
| | Eye Corrosion | **0.983** | 0.904 | **0.957** | 0.798 | **0.908** | 0.619 | **0.965** | 0.723 | **0.944** | 0.904 |
| | FDAMDD | 0.804 | **0.874** | 0.736 | **0.988** | 0.471 | **0.804** | 0.734 | **0.999** | **0.737** | 0.692 |
| | NR-AhR | **0.943** | 0.923 | **0.862** | 0.857 | 0.573 | **0.714** | 0.858 | **0.906** | **0.896** | 0.801 |
| | NR-AR-LBD | **0.915** | 0.900 | **0.936** | 0.827 | 0.472 | **0.634** | **0.942** | 0.822 | 0.783 | **0.836** |
| | NR-AR | **0.886** | 0.874 | 0.890 | **0.988** | 0.348 | **0.804** | 0.896 | **0.999** | **0.731** | 0.692 |
| | NR-Aromatase | 0.852 | **0.912** | **0.849** | 0.823 | 0.264 | **0.647** | **0.859** | 0.810 | 0.615 | **0.836** |
| | NR-ER-LBD | 0.850 | **0.938** | **0.903** | 0.900 | 0.364 | **0.441** | **0.918** | 0.906 | 0.618 | **0.788** |
| | NR-ER | 0.771 | **0.904** | **0.815** | 0.798 | 0.320 | **0.619** | **0.845** | 0.723 | 0.567 | **0.904** |
| | NR-PPAR-gamma | 0.893 | **0.912** | **0.896** | 0.823 | 0.344 | **0.647** | **0.901** | 0.810 | 0.750 | **0.836** |
| | Skin Sensitization | 0.707 | **0.938** | 0.775 | **0.900** | **0.462** | 0.441 | 0.539 | **0.906** | **0.889** | 0.788 |
| | SR-ARE | 0.863 | **0.874** | 0.827 | **0.988** | 0.469 | **0.804** | 0.850 | **0.999** | **0.701** | 0.692 |
| | SR-ATAD5 | 0.874 | **0.923** | **0.919** | 0.857 | 0.361 | **0.714** | **0.929** | 0.906 | 0.640 | **0.801** |
| | SR-HSE | **0.907** | 0.874 | 0.868 | **0.988** | 0.393 | **0.804** | 0.875 | **0.999** | **0.750** | 0.692 |
| | SR-MMP | 0.927 | **0.938** | 0.897 | **0.900** | **0.660** | 0.441 | **0.908** | 0.906 | **0.835** | 0.788 |
| | SR-p53 | 0.881 | **0.938** | 0.841 | **0.900** | 0.365 | **0.441** | 0.849 | **0.906** | 0.723 | **0.788** |
| | Average | 0.865 | **0.909** | 0.835 | **0.865** | 0.501 | **0.646** | 0.828 | **0.863** | 0.764 | **0.809** |

Table S11: Comparison of model parameters, training time, and performance in Task 7 of Activity-33.

| Data Type | Methods | Parameter (KB) | Time (m) | $r^2$ (↑) | RMSE (↓) | Ligand optimization |
|---|---|---|---|---|---|---|
| Fingerprint-based | RF | 127 | 0.0049 | 0.2071 | 0.8081 | ✗ |
| | MLP | 263 | 0.82 | 0.1775 | 0.8231 | ✗ |
| Graph-based | GATs | 8 | 1.12 | 0.2871 | 2.9244 | ✗ |
| | GINs | 8,345 | 3.87 | 0.0360 | 1.3547 | ✗ |
| | Neural FP | 27 | 3.08 | 0.0437 | 0.8868 | ✗ |
| | Weave | 62 | 1.47 | 0.2871 | 0.8604 | ✗ |
| | MPNN | 66 | 8.28 | 0.0008 | 2.4965 | ✗ |
| | Attentive FP | 13 | 8.70 | 0.1866 | 0.9050 | ✗ |
| | Uni-Mol | 47,594 | 4.13 | 0.2770 | 2.1393 | ✗ |
| | AFSE | 242 | 10.20 | 0.5079 | 0.8799 | ✗ |
| | OLB-AC (Ours) | 520 | 18.67 | **0.5842** | **0.7754** | ✓ |

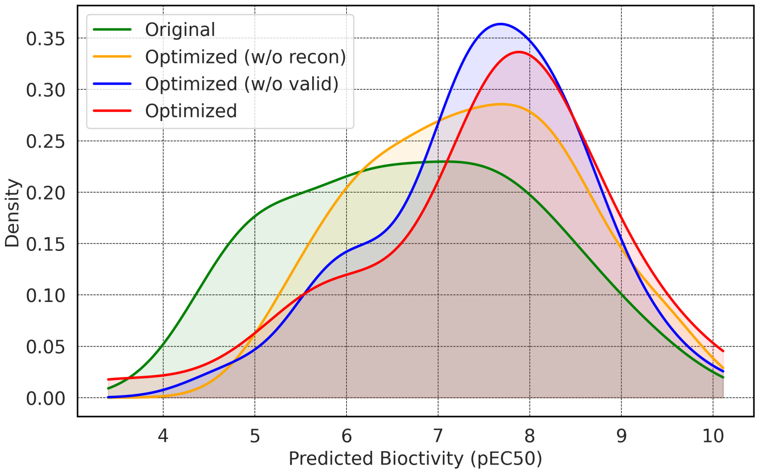


Figure S1: The predicted bioactivity distributions between optimized molecules and the original low-activity molecules in Task 7 of Activity-33. In the legend, ”w/o-recon” or ”w/o-valid” denotes that the original molecule was optimized through model training without reconstruction loss or validity loss, respectively.

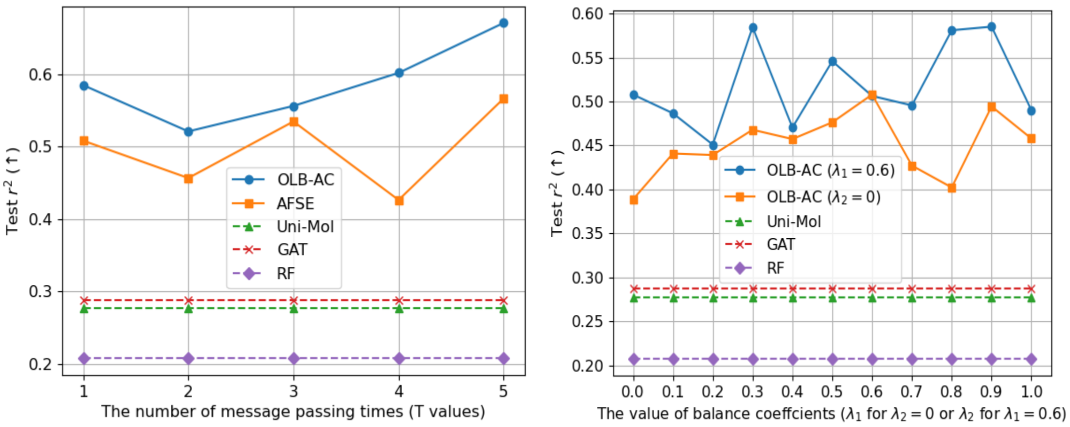


(a) $T$ values

(b) $\lambda_1$ and $\lambda_2$

Figure S2: Ablation study on hyperparameters in Task 7 of Activity-33. The upward arrow ”↑” in the y-axis label denotes that higher is better. The performance of Uni-Mol, GAT, and RF are used as baselines (dashed lines).

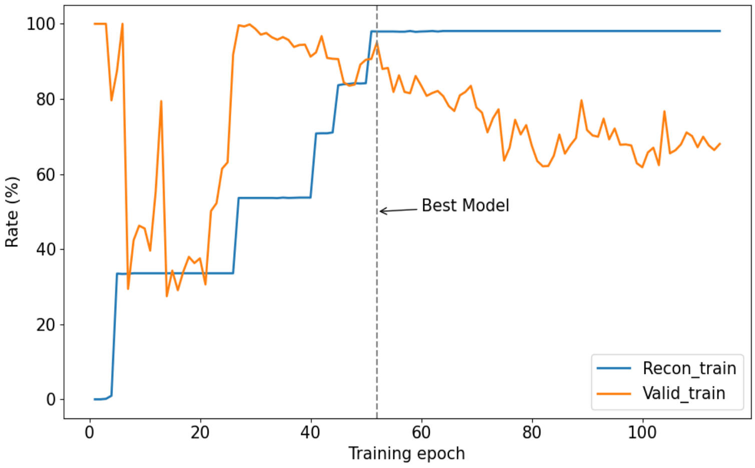


Figure S3: The reconstruction (“Recon_train”) rate and validity (“Valid_train”) rate during training epochs in Task 12 of Activity-33. The “Best Model” was selected at the highest value on the sum of reconstruction and validity rates.

**Algorithm S1** Molecule embedding algorithm of Attentive FP

**Input:** molecule $\mathbf{m}$, the step size $L$ of the message passing of the graph neural network, the number of steps $T$ for aggregating molecular features.

**Output:** Molecular embeddings $\mathbf{f}$. Initialization: $l \leftarrow 0$,$t \leftarrow 0$

1: Extract chemical features $\mathbf{a}_i$ and $\mathbf{b}_{i,j}$ for each atom and bond from $\mathbf{m}$, where $i$ and $j$ are atomic numbers

2: Obtain the initially hidden feature of each atom $\mathbf{a}_i$:

$$\mathbf{h}_i^0 = \mathbf{W}_0 \cdot \mathbf{a}_i + \mathbf{c}_0$$

3: Get the chemical features of the adjacent atoms $\mathbf{a}_j,\ j \in N(i)$ and their bonds $\mathbf{b}_{i,j}$:

$$\mathbf{h}_{ji}^0 = \text{leaky\_relu}(\mathbf{W}_1 \cdot [\mathbf{a}_j, \mathbf{b}_{i,j}] + \mathbf{c}_1)$$

4: **while** $l < L$ **do**

5: Get the attention weight between each atom and its adjacent atoms:

$$w_{ji} = \text{softmax}(\text{leaky\_relu}(\mathbf{W}_2 \cdot \text{dropout}([\mathbf{h}_i^l, \mathbf{h}_{ji}^l]) + \mathbf{c}_2))$$

6: Get the context feature of each atom:

$$\mathbf{C}_i^l = \text{elu}(\sum_j w_{ji} \cdot \mathbf{W}_3(\text{dropout}(\mathbf{h}_{ji}^l)) + \mathbf{c}_3)$$

7: Readout the next hidden feature of each atom:

$$\mathbf{h}_i^{l+1} = \text{relu}(\text{GRU}(\mathbf{C}_i^l, \mathbf{h}_i^l))$$

8: $l \leftarrow l + 1$

9: Update the adjacent features for each atom:

$$\mathbf{h}_{ji}^l = \text{leaky\_relu}(\mathbf{W}_4 \cdot \text{dropout}([\mathbf{h}_i^{l-1}, \mathbf{h}_{ji}^{l-1}]) + \mathbf{c}_4)$$

10: **end while**

11: Aggregate the hidden features of all atoms to get the molecular feature:

$$\mathbf{h}^L = \sum_i \mathbf{h}_i^L$$

12: Let molecules be the supernodes: $\mathbf{h}^0 \leftarrow \mathbf{h}^L$

13: Let all atoms be the adjacent nodes: $\mathbf{h}_N^0 \leftarrow [\mathbf{h}_1^L, \mathbf{h}_2^L, \cdots, \mathbf{h}_{N_a}^L]$

14: **while** $t \leq T$ **do**

15: Obtain molecular hidden features $\mathbf{h}^t$ by performing steps 5-9 with $\mathbf{W}_5$, $\mathbf{W}_6$, $\mathbf{W}_7$ and $\mathbf{b}_5$, $\mathbf{b}_6$, $\mathbf{b}_7$

16: $t \leftarrow t + 1$

17: **end while**

18: Obtain the molecular embedding $\mathbf{f} = \mathbf{h}^T$

**Algorithm S2** Molecule graph generation algorithm of AGRNs

**Input:** Attentive FP $\mathbf{f}$, the change of feature $\mathbf{d}$ ($\mathbf{d} = 0$ for reconstruction, and $\mathbf{d} \neq 0$ for generation), the step size $L$ of the message passing of the graph neural network, the number of steps $T$ for aggregating molecular features, and the hidden features of each atom $\mathbf{h}_i^L$.

**Output:** Chemical features $\mathbf{a}_i$ and $\mathbf{b}_{i,j}$ for each atom and bond in the molecule $\mathbf{m}$, where $i$ and $j$ are atoms 's number.

Initialization: $t \leftarrow T, l \leftarrow L$

Calculate the component of the molecular feature on each atom:

$$\gamma_i = \frac{\exp(\langle \mathbf{f} + \mathbf{d}, \mathbf{h}_i^L \rangle)}{\sum_j \exp(\langle \mathbf{f} + \mathbf{d}, \mathbf{h}_j^L \rangle)}$$

Obtain the hidden features of each atom used to reconstruct the molecule:

$$\mathbf{g}_i^T = \gamma_i(\mathbf{f} + \mathbf{d}) + \mathbf{h}_i^L$$

**while** $t \geq 1$ **do**

  Get the relationship information $\mathbf{r}_i^t$ between the molecule and its atoms:

$$\mathbf{r}_i^t = \text{elu}(\mathbf{W}_1 \cdot (\text{dropout}([\mathbf{f} + \mathbf{d}, \mathbf{g}_i^t])) + \mathbf{c}_1)$$

  Deduce the hidden feature on each atom: $\mathbf{g}_i^{t-1} = \text{relu}(\text{GRU}(\mathbf{r}_i^t, \mathbf{g}_i^t))$

  $t \leftarrow t - 1$

**end while**

Assigning atom features: $\mathbf{g}^L \leftarrow \mathbf{g}_i^{t=0}$

Assigning adjacent features: $\mathbf{g}_{ji}^L \leftarrow \mathbf{g}_j^{t=0}$

**while** $l \geq 1$ **do**

  Get the attention weight between each atom and its adjacent atoms

$$w_{ji} = \text{softmax}(\text{leaky\_relu}(\mathbf{W}_2 \cdot \text{dropout}([\mathbf{g}_i^l, \mathbf{g}_{ji}^l]) + \mathbf{c}_2))$$

  Get the context feature of each atom:

$$\mathbf{C}_i^l = \text{elu}(\sum_j w_{ji} \cdot \mathbf{W}_3(\text{dropout}(\mathbf{g}_{ji}^l)) + \mathbf{c}_3)$$

  Deduce the hidden feature on each atom: $\mathbf{g}_i^{l-1} = \text{relu}(\text{GRU}(\mathbf{C}_i^l, \mathbf{g}_i^l))$

  $l \leftarrow l - 1$

  Update the adjacent features for each atom:

$$\mathbf{g}_{ji}^l = \text{leaky\_relu}(\mathbf{W}_4 \cdot \text{dropout}([\mathbf{g}_i^{l+1}, \mathbf{g}_{ji}^{l+1}]) + \mathbf{c}_4)$$

**end while**

Deduce the chemical feature of each atom: $\widetilde{\mathbf{a}}_i = \phi_a(\mathbf{W}_4 \cdot \mathbf{g}_i^{l=0} + \mathbf{c}_4)$

Deduce the chemical features of each bond:

$$\widetilde{\mathbf{b}}_{i,j} = \phi_b(\text{leaky\_relu}(\mathbf{W}_5 \cdot \text{dropout}([\mathbf{g}_i^{l=0}, \mathbf{g}_j^{l=0}]) + \mathbf{c}_5))$$

**Algorithm S3** Optimizing ligand bioactivity through OLB-AC

**Input:** Attentive FP $\mathbf{f}$ and the hidden feature $\mathbf{h}_i^r$ of each atom $\mathbf{a}_i$ on step $L$.

**Output:** Generated chemical features of atoms and bonds $\widetilde{\mathbf{a}}_i$ and $\widetilde{\mathbf{b}}_{i,j}$, where $i$ and $j$ are adjacent atoms's number.

1: Generate random unit vector $\mathbf{r}_0 \in \mathbb{R}^{d_f}$ using i.i.d. Gaussian distributions, where $d_f$ is the dimension of $\mathbf{f}$.

2: Calculate $\mathbf{d}$ via taking the gradient of $D$ with respect to $\mathbf{r}$ on $\mathbf{f}$ at $\varepsilon\mathbf{r}_0$:

$$\mathbf{g} \leftarrow \nabla_{\mathbf{r}} D(\mathbf{W}[\mathbf{f},\mathbf{f}], \mathbf{W}[\mathbf{f},\mathbf{f} \oplus \mathbf{r}])|_{\mathbf{r}=\varepsilon\mathbf{r}_0}$$

$$\mathbf{d} \leftarrow \eta \frac{\mathbf{g}}{\|\mathbf{g}\|_2}$$

$$D(\mathbf{W}[\mathbf{f},\mathbf{f}], \mathbf{W}[\mathbf{f},\mathbf{f} \oplus \mathbf{r}]) \triangleq (\sigma(\frac{\mathbf{W}[\mathbf{f},\mathbf{f}+\mathbf{r}]}{\mathbf{W}[\mathbf{f},\mathbf{f}]}) - \gamma)^2$$

$$+(\sigma(\frac{\mathbf{W}[\mathbf{f},\mathbf{f}-\mathbf{r}]}{\mathbf{W}[\mathbf{f},\mathbf{f}]}) - \gamma)^2$$

$$\sigma(x) \triangleq \mathrm{Sigmoid}(x) = 1/(1+e^{-x})$$

$$\gamma = \mathrm{Sigmoid}(1)$$

3: Execute Algorithm S2 to generate the atom and bond features of the molecule near the activity cliff:

$$[\widetilde{\mathbf{a}}_i, \widetilde{\mathbf{b}}_{i,j}] = \mathrm{AGRNs}(\mathbf{f}+\mathbf{d})$$

**Text S1: Performance Indexes**

**1) The square of Pearson correlation coefficient** ($r^2$) index is adopted for evaluating the performance of activity prediction from participants during the Kaggle 2012, which is defined as

$$r^2 = \frac{\left[\sum_{i=1}^{n}\left(y_i - \bar{y}\right)\left(\hat{y}_i - \bar{y}\right)\right]^2}{\sum_{i=1}^{n}\left(y_i - \bar{y}\right)^2 \sum_{i=1}^{n}\left(\hat{y}_i - \bar{\hat{y}}\right)^2}, \quad (1)$$

where $y_i$ and $\hat{y}_i$ are the true and predicted bioactivity values, respectively, and $\bar{y}$ and $\bar{\hat{y}}_i$ are the mean values of the true and predicted bioactivity, respectively. $n$ is the total number of test samples. The larger the $r^2$, the higher the overall accuracy of the model prediction.

**2)The Root Mean Square Error (RMSE)** is a widely used performance index for evaluating the performance of regression models. It is defined as the square root of the average of the squared differences between the predicted and actual values as follows:

$$\text{RMSE} = \sqrt{\frac{1}{n}\sum_{i=1}^{n}\left(y_i - \hat{y}_i\right)^2}, \quad (2)$$

where $y_i$ and $\hat{y}_i$ are the true and predicted bioactivity values, respectively, and $n$ is the total number of test samples. The lower the RMSE, the better the performance of the regression model.

**3) The area under the receiver operating characteristic curve (AUC).** The Receiver Operating Characteristic (ROC) curve is a popular method for evaluating the performance of binary classifiers. The area under the ROC curve (AUC) is a scalar value between 0 and 1, where an AUC value of 1 indicates a perfect classifier, and an AUC value of 0.5 indicates random chance.

**4) Accuracy (ACC)** is the ratio of the number of correct predictions to the total number of predictions. It is defined as:

$$\text{ACC} = \frac{TP + TN}{TP + TN + FP + FN}, \quad (3)$$

where $TP$ (True Positives) are the number of instances that are correctly classified as positive, $TN$ (True Negatives) are the number of instances that are correctly classified as negative, $FP$ (False Positives) are the number of instances that are incorrectly classified as positive, and $FN$ (False Negatives) are the number of instances that are incorrectly classified as negative. This index provides an overall measure of the accuracy of a model, but it can be misleading in cases where the class distribution is imbalanced.

**5) The Matthews Correlation Coefficient (MCC)** is a more robust performance index that takes into account both the accuracy and the imbalance of the classes. It is defined as:

$$\text{MCC} = \frac{TP \cdot TN - FP \cdot FN}{\sqrt{(TP + FP)(TP + FN)(TN + FP)(TN + FN)}}, \quad (4)$$

where positive and negative classes are equally balanced, a value of 1 indicates a perfect classifier, a value of 0 indicates a random classifier, and a value of -1 indicates a classifier that performs worse than random.

**6) Specificity**, also known as recall, is the ratio of the number of true positive predictions to the number of actual positive instances. It is defined as:

$$\text{Sensitivity} = \frac{TP}{TP + FN}. \tag{5}$$

Specificity is a measure of the classifier's ability to identify negative instances.

**7) Sensitivity**, also known as the True Positive Rate (TPR), is the ratio of the number of true negative predictions to the number of actual negative instances. It is defined as:

$$\text{Specificity} = \frac{TN}{TN + FP}. \tag{6}$$

Sensitivity is a measure of the classifier's ability to identify positive instances.

**Text S2: Enhancing inhibitory efficacy and reducing toxicity through OLB-AC's ligand bioactivity optimization**

*1) Datasets and settings:* This section involves three inhibitor datasets and three toxicity datasets in ADMET-25, namely CYP1A2, CYP2C9, CYP3A4 inhibitor datasets, and NR-AhR, NR-ER, AMES Toxicity datasets. These datasets are characterized by relatively abundant MMP-Cliffs compared to other datasets in ADMET-25.

Initially, we identify MMP-Cliffs within the typical datasets, where ligands differ by only one atom element, transitioning from non-inhibitor to inhibitor or from highly toxic to non-toxic. Subsequently, we designate the remaining ligands as the training set. Next, we reintroduce the MMP-Cliffs ligands with non-inhibitor or strong toxicity back into the training set. We then observe whether the ligands optimized by OLB-AC exhibit inhibitory properties or remain non-toxic.

*2) Evaluation methods:* We assess the generated molecules based on molecular property indexes, including toxicity (high or non-toxic) and inhibition status (yes or no), as well as the same properties as those used in bioactivity optimization, namely quantitative estimate of drug-likeness (QED), synthetic accessibility (SA), and octanol-water partition coefficient (LogP).

*3) Results:* In Supplementary Table S5, we provide representative results of OLB-AC optimization, focusing on converting non-inhibitor ligands to inhibitor MMP-Cliffs ligands. The term "Anchor Ligands" in the table refers to the non-inhibitor ligands in the training set, while "Optimized Ligands" represents the inhibitor ligands generated by OLB-AC based on these "Anchor Ligands". The calculated properties, including QED, SA, and LogP, further support the optimization results, indicating that the optimized inhibitor ligands can exhibit improved drug-like properties, increased synthesis feasibility, and enhanced drug absorption compared to the non-inhibitor ligands.

In Supplementary Table S6, we present the results of OLB-AC optimization targeting toxic to non-toxic MMP-Cliff ligands. In this table, "Anchor Ligands" refer to toxic ligands in the training set, while "Optimized Ligands" denote non-toxic ligands optimized by OLB-AC based on the "Anchor Ligands". Across multiple datasets, OLB-AC demonstrates the capability to optimize ligand toxicity, resulting in improved drug-like properties, enhanced synthesis feasibility, and better drug absorption for the majority of the ligands.

# References

[1] Yueming Yin, Haifeng Hu, Zhen Yang, Feihu Jiang, Yihe Huang, and Jiansheng Wu. Afse: towards improving model generalization of deep graph learning of ligand bioactivities targeting gpcr proteins. *Briefings in Bioinformatics*, 23(3):bbac077, 2022.

[2] Guoli Xiong, Zhenxing Wu, Jiacai Yi, Li Fu, Zhijiang Yang, Changyu Hsieh, Mingzhu Yin, Xiangxiang Zeng, Chengkun Wu, Aiping Lu, et al. Admetlab 2.0: an integrated online platform for accurate and comprehensive predictions of admet properties. *Nucleic Acids Research*, 49(W1):W5–W14, 2021.
